# Chip-based analysis of exosomal mRNA mediating drug resistance in glioblastoma

**DOI:** 10.1038/ncomms7999

**Published:** 2015-05-11

**Authors:** Huilin Shao, Jaehoon Chung, Kyungheon Lee, Leonora Balaj, Changwook Min, Bob S. Carter, Fred H. Hochberg, Xandra O. Breakefield, Hakho Lee, Ralph Weissleder

**Affiliations:** 1 Center for Systems Biology, Massachusetts General Hospital, 185 Cambridge St, CPZN 5206, Boston, Massachusetts 02114, USA; 2 Department of Neurology, Massachusetts General Hospital, Fruit St, Boston, Massachusetts 02114, USA; 3 Program in Neuroscience, Harvard Medical School, 200 Longwood Ave, Boston, Massachusetts 02115, USA; 4 Division of Neurological Surgery, UCSD School of Medicine, San Diego, California 92103, USA; 5 Massachusetts General Hospital Cancer Center, Boston, Massachusetts 02114, USA; 6 Department of Radiology, Massachusetts General Hospital, Fruit St, Boston, Massachusetts 02114, USA; 7 Department of Systems Biology, Harvard Medical School, 200 Longwood Ave, Boston, Massachusetts 02115, USA

## Abstract

Real-time monitoring of drug efficacy in glioblastoma multiforme (GBM) is a major clinical problem as serial re-biopsy of primary tumours is often not a clinical option. MGMT (O^6^-methylguanine DNA methyltransferase) and APNG (alkylpurine-DNA-N-glycosylase) are key enzymes capable of repairing temozolomide-induced DNA damages and their levels in tissue are inversely related to treatment efficacy. Yet, serial clinical analysis remains difficult, and, when done, primarily relies on promoter methylation studies of tumour biopsy material at the time of initial surgery. Here we present a microfluidic chip to analyse mRNA levels of MGMT and APNG in enriched tumour exosomes obtained from blood. We show that exosomal mRNA levels of these enzymes correlate well with levels found in parental cells and that levels change considerably during treatment of seven patients. We propose that if validated on a larger cohort of patients, the method may be used to predict drug response in GBM patients.

## Abstract (web summary)

Predicting and monitoring chemotherapy response remains a challenge for glioma treatment. Here the authors show that a microfluidic device can isolate glioma-derived exosomes from patient blood and accurately determine the levels of mRNA of key enzymes important for chemoresponsiveness.

## Graphical abstract

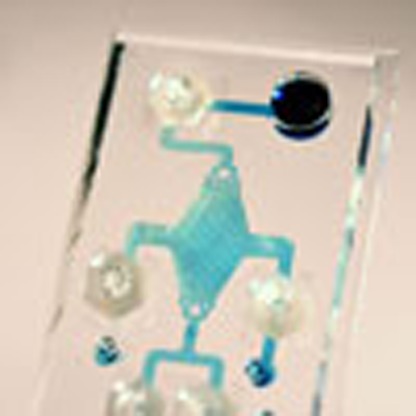

Glioblastoma multiforme (GBM) is the most common primary malignancy of the central nervous system. Currently, standard GBM treatments include maximal safe surgical resection, radiation and adjuvant temozolomide (TMZ) chemotherapy^1^. The introduction of TMZ, in particular, has increased overall survival from 12.1 months to 14.6 months. Despite these approaches, overall response remains poor. Not all tumours respond to TMZ and drug efficacy varies during treatment. Elevated promoter DNA methylation of drug resistance genes has been shown to enhance TMZ response in GBM patients, by reducing the expression of these nuclear proteins in cells^2^,^3^. Longitudinal testing for drug response and resistance of the tumour, however, is done infrequently because of the complexities and morbidity of performing repeat biopsies. There is thus a critical need for minimally invasive biomarkers to objectively measure response during therapeutic interventions. Prior research has shown that extracellular vesicles, including exosomes, can be readily harvested from blood for further analysis and thus represent an attractive source of tumour-derived materials^4^,^5^,^6^.

Exosomes are membrane-bound phospholipid nanovesicles (50–200 nm in diameter) actively secreted by mammalian cells and, in particular, dividing tumour cells^7^,^8^. They are abundant (>10^9^ vesicles ml^−1^ in serum), stable and contain unique proteins and nucleic acids reflective of their cells of origin^4^,^9^,^10^. Beyond their size and density (which are often used for isolation), exosomes are also enriched with specific membrane markers (CD63, CD81, ALIX)^9^. Moreover, GBM-derived exosomes can be differentiated from host exosomes by epidermal growth factor receptor (EGFR) amplification and specific mutations such as EGFRvIII deletion^4^,^11^,^12^,^13^. These identifying features enable affinity enrichment of cancer-specific exosome populations in a laboratory setting but have not been adapted to the clinic. We have previously described two nanotechnology-inspired biosensing platforms for point-of-care analysis of exosome proteins^12^,^14^. These technologies were primarily developed for diagnostic rather than prognostic purposes, the focus of the current study. Given the clinical need to detect the emergence of drug resistance during therapy, we started to look for intra-exosomal proteins that play key roles in drug resistance pathways^15^,^16^. However, this proved challenging with previous technologies, presumably because many of these proteins are compartmentalized in the nucleus and are therefore variably partitioned into exosomes. We thus argued that the mRNA counterparts of these nuclear proteins that are translated in the cytoplasm could be more readily detected within exosomes.

In the current study, we describe a sensitive and comprehensive microfluidic platform termed immuno-magnetic exosome RNA (iMER) analysis, which enables enrichment of cancer-specific exosomes from blood and fast, on-chip analysis of their RNA contents. The iMER system integrates immunomagnetic selection, RNA collection and real-time PCR into a single microfluidic chip format. Using this technology, we compared the mRNA profiles of GBM-derived exosomes against those of their cells of origin and followed dynamic sequential changes on treatment initiation. The study identified key exosomal mRNA markers potentially predictive of TMZ resistance and showed capacity of exosomal RNA analysis for probing the epigenetic status of the primary tumour. Furthermore, we analysed clinical blood samples from patients with confirmed GBM, and showed that exosomal mRNA profiles could be correlated to treatment response, independent of the initial epigenetic status in tissue biopsy.

## Results

### iMER platform

**Figure 1:**
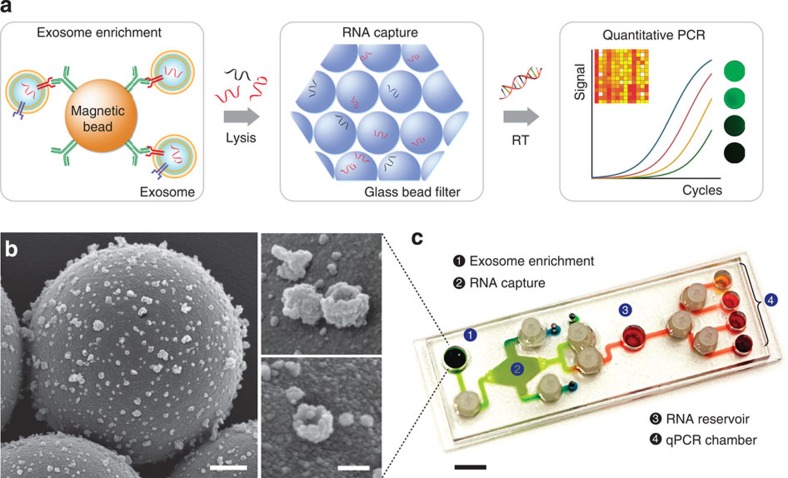
The immunomagnetic exosomal RNA (iMER) platform. (**a**) The iMER platform is designed to enable exosome enrichment, RNA extraction, reverse transcription and real-time analyses of distinct RNA targets in one small device. Cancer exosomes in serum are first captured onto magnetic microbeads containing affinity ligands (for example, anti-CD63 and anti-EGFR). The immuno-enriched exosomal population is then lysed and the lysate flows through a glass bead filter, where RNA efficiently adsorbs onto packed glass beads. Finally, the collected RNA is eluted and reverse-transcribed for real-time amplification and quantitation. (**b**) Scanning electron micrographs of magnetic microbeads after immunoaffinity capture. Microbeads (left, 3 μm) functionalized with antibodies against EGFRvIII, a cancer-specific deletion mutant, captured innumerable tumour vesicles from GLI36vIII conditioned medium. High-magnification micrographs (right) show that many of the captured vesicles exhibit cup-shaped characteristic of exosomes. Scale bars, 500 nm, 100 nm (inset). (**c**) Photograph of the microfluidic iMER prototype. The cartridge was developed to house all components of the iMER procedure, including (1) an immunomagnetic capture site, (2) a filter composed of densely packed glass beads for RNA extraction, (3) a main reservoir for reverse transcription and (4) qPCR chambers for multiplexed detection. Scale bar, 1 cm.

The iMER platform integrates three functional compartments: targeted enrichment of extracellular vesicles, on-chip RNA isolation and real-time RNA analysis (Fig. 1a). The enrichment step immunomagnetically separates cancer exosomes from host-derived exosomes so that subsequent analyses are performed on enriched populations. The collected exosomes are then lysed, and the lysate is passed through a glass-bead filter. During this process, RNA is adsorbed onto glass beads via electrostatic interactions. RNA is subsequently eluted and reverse-transcribed for detection by quantitative polymerase chain reaction (qPCR).

We optimized the iMER assay for GBM-specific exosome analysis. To enrich for and capture GBM-derived exosomes, we functionalized magnetic microbeads (3 μm in diameter) with antibodies against EGFR (recognizing both EGFR wild-type and EGFRvIII mutation). When beads were incubated with GBM-derived exosomes, the entire surface appeared densely covered (Fig. 1b). Within these isolated vesicles, a large amount of mRNA was found, including mRNA of nuclear proteins (Supplementary Fig. 1). To streamline the downstream mRNA analysis, we prepared a microfluidic cartridge for subsequent steps (Fig. 1c). Specifically, four key components were integrated onto the chip: (i) an immunomagnetic capture site for exosome enrichment, (ii) a filter chamber packed with glass beads for RNA isolation and elution, (iii) a reservoir for reverse transcription and pre-amplification of rare targets and (iv) multiple qPCR chambers for target mRNA detection. The fluidic flow was controlled through torque-activated valves^17^,^18^ (see Supplementary Fig. 2 for device operation and dimensions). The cartridge was loaded into a custom-designed PCR system equipped with a thermal cycler and a fluorescence detector.

### Exosome enrichment and RNA analysis

**Figure 2:**
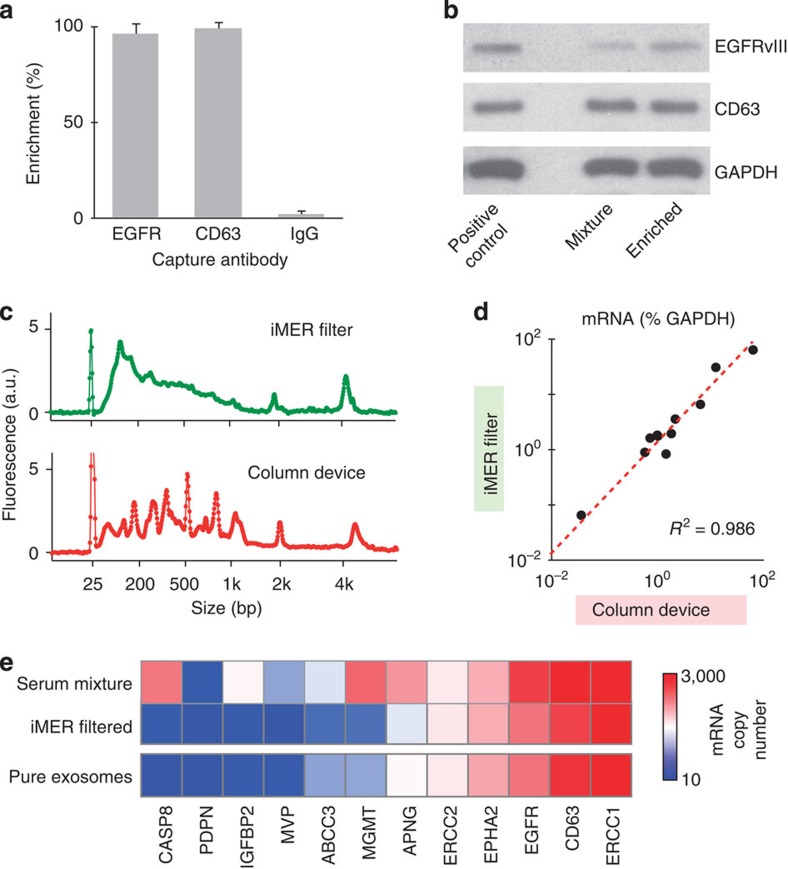
Enrichment and analysis. (**a**) Exosome enrichment efficiency. Exosomes from SKMG3 cells were incubated with magnetic immunobeads (functionalized with either anti-EGFR, CD63 or IgG antibodies). Following magnetic separation, the achieved enrichment efficiency was determined. Note the high capture efficiency (>95%) for anti-EGFR and anti-CD63 microbeads as opposed to the IgG control (<2%). (**b**) Exosome enrichment specificity. Exosomes from EGFRvIII-positive GLI36vIII cells and negative GBM20/3 cells were mixed in 1:1 ratio (‘mixture') and subjected to EGFR-specific immunomagnetic isolation. As compared with equal amounts of GLI36vIII exosomes (‘positive control'), western blotting on the isolated exosomes (‘enriched') confirmed the enrichment of specific targets from a mixture of exosomes of different origins. (**c**) Comparison of RNA extraction. iMER-extracted RNA showed better structural integrity and quality profile to that extracted by a commercially available column (Qiagen). a.u.=arbitrary unit. (**d**) Correlation between iMER-extracted and column-extracted mRNA. The abundance of mRNA targets measured by iMER and a commercial system matched well (*R*^2^=0.986). All analyses were normalized against GAPDH. (**e**) iMER enrichment in serum. SKMG3 exosomes were spiked into normal human serum (‘serum mixture'). After targeted EGFR enrichment, captured exosomes were lysed and analysed on-chip (‘iMER filtered'). All measurements were performed in triplicate and the data are displayed as mean values.

We first evaluated the performance of two operations, key to accurate diagnostics: exosome enrichment and RNA isolation. Exosomes harvested from GBM cell culture (SKMG3 and GLI36vIII) were incubated with immunomagnetic microbeads. Following magnetic separation, we determined the capture efficiency by measuring the amount of target proteins in the supernatant using chemiluminescence ELISA. The iMER assay showed capture efficiencies of >93% for different targets (Fig. 2a and Supplementary Fig. 3). Off-target binding was <5%, which was determined using microbeads functionalized with IgG control antibodies (Supplementary Fig. 3). Western blotting on isolated exosomes via EGFR-specific capture further confirmed the capacity of the system to enrich and retrieve specific targets from a mixture of exosomes of different origins (Fig. 2b and Supplementary Fig. 4).

To determine the RNA isolation yield, enriched exosomes from GLI36vIII culture were lysed on chip. Lysate aliquots were then processed for RNA extraction by the iMER cartridge as well as by a commercially available column filter (Qiagen). For a given sample volume, the iMER cartridge had 50% higher isolation yield compared with the column filter (Supplementary Fig. 5). This could be attributed to the iMER's high surface area of densely packed glass beads. Collected RNA retained structural integrity over a broad range of sizes (Fig. 2c) as determined by electrophoresis analysis (2,100 Bioanalyzer, Agilent). We further performed RT-qPCR analyses on iMER-extracted and column-extracted samples. The abundance of mRNA targets measured by iMER and in column-RNA using a commercial system matched well (*R*^2^=0.986), which indicated unbiased RNA extraction and amplification by the iMER platform (Fig. 2d).

Combining on-chip exosome enrichment and RNA isolation, the iMER assay was able to detect specific mRNA signatures. We prepared a mock clinical sample by spiking SKMG3 exosomes (∼10^8^ ml^−1^) into normal human sera. Direct mRNA profiling of the serum mixture showed poor correlation to the signatures from pure cancer exosomes (*R*^2^=0.693). Conversely, with the iMER enrichment and detection, we observed excellent correlation (*R*^2^=0.951) between pure exosomes and serum samples (Fig. 2e). In comparison with standard procedure, which entails hours of ultracentrifugation and RNA extraction from a large sample volume (>1 ml), the entire iMER analysis (from exosome enrichment to RT-qPCR) was completed within ∼2 h, and required a sample volume of ∼100 μl for multiplexed analysis.

### mRNA profiles of exosomes and parental cells

**Figure 3:**
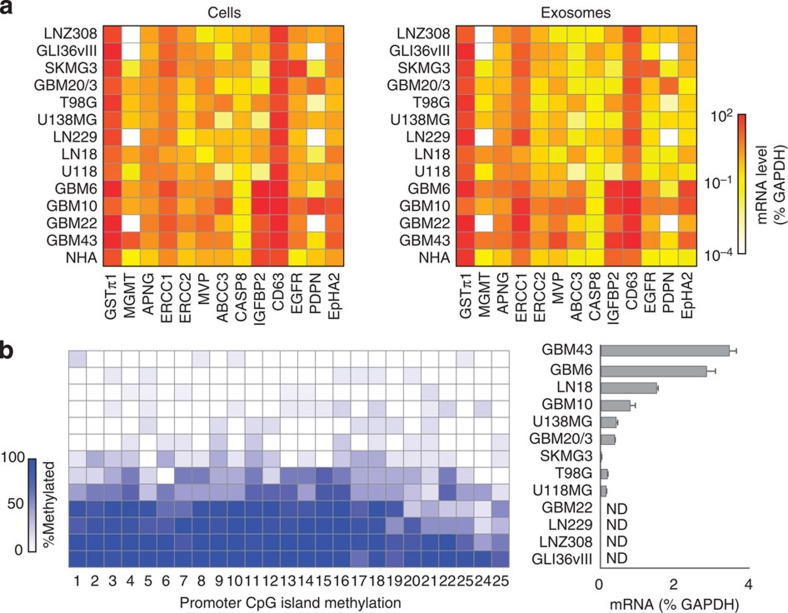
Exosome mRNA analysis of 14 cell lines. (**a**) mRNA drug resistance markers (GSTπ1, MGMT, APNG, ERCC1, ERCC2, MVP, ABCC3, CASP8 and IGFBP2), exosome marker (CD63) and representative diagnostic markers (EGFR, PDPN and EPHA2) were profiled in both parental cells (left, by conventional qRT-PCR) and corresponding exosomes (right, by iMER). mRNA quantities were normalized to intrinsic GAPDH mRNA. Note the excellent correlation between exosomal mRNA markers and those found in parental cells (*R*^2^=0.918). (**b**) Comparison of exosomal MGMT mRNA analysis with MGMT promoter DNA methylation in parental cells. The numbers on the *x*-axis (left) refer to the specific CpG islands in the MGMT DNA promoter sequence. There is an inverse correlation between promoter DNA methylation in the parental cells (left, determined via bisulfite sequencing for the extent of methylation at different CpG islands) and levels of exosomal MGMT mRNA (right). ND=non-detectable. All measurements were performed in triplicate and the data are shown as mean±s.d.

We next examined whether GBM-derived exosomes assume similar mRNA profiles to those found in their originating cells. On the basis of previous reports, three categories of mRNA targets were selected: (i) diagnostic markers—EGFR^11^, podoplanin (PDPN)^19^, ephrin type-A receptor 2 (EPHA2)^20^; (ii) drug resistance markers—O^6^-methylguanine DNA methyltransferase (MGMT)^2^, alkylpurine-DNA-N-glycosylase (APNG)^3^, glutathione S-transferase (GSTπ1)^21^, excision repair cross-complementation (ERCC1^22^ and ERCC2^23^,^24^), major vault protein (MVP)^25^,^26^, ATP-binding cassette/multidrug resistance-associated protein 3 (ABCC3)^27^, caspase-8 (CASP8)^28^ and insulin-like growth factor binding protein 2 (IGFBP2)^29^; and (iii) a generic exosome marker—CD63^9^,^30^. A panel of human GBM cell lines and a control (normal human astrocytes; NHA) were cultured. Cellular mRNA contents were measured by conventional RT-qPCR (Fig. 3a, left); the corresponding exosomal mRNA were profiled by the iMER platform (Fig. 3a, right). The mRNA profiles between cells and exosomes showed high correlation (*R*^2^=0.918; Supplementary Fig. 6).

We also compared the exosomal mRNA levels with promoter DNA methylation status in tumour cells. DNA promoter methylation of drug resistance gene (MGMT) reduces the expression of the nuclear protein in cells, and thereby enhances drug efficacy in GBM patients^2^. We reasoned that exosomal mRNA could be used as a surrogate indicator of DNA methylation status. Indeed, a high level of MGMT promoter methylation in parent cells correlated inversely with low MGMT mRNA levels in exosomes and vice versa (Fig. 3b). We further observed a match between cellular protein expression and exosomal mRNA levels in both GBM cell lines (Supplementary Figs 7 and 8) and mouse models (Supplementary Fig. 9). In mouse xenograft models of human GBM, the level of human MGMT mRNA in serum exosomes was elevated in mice bearing MGMT-positive tumour (GBM10).

### Exosomal mRNA as a predictive marker for treatment efficacy

**Figure 4:**
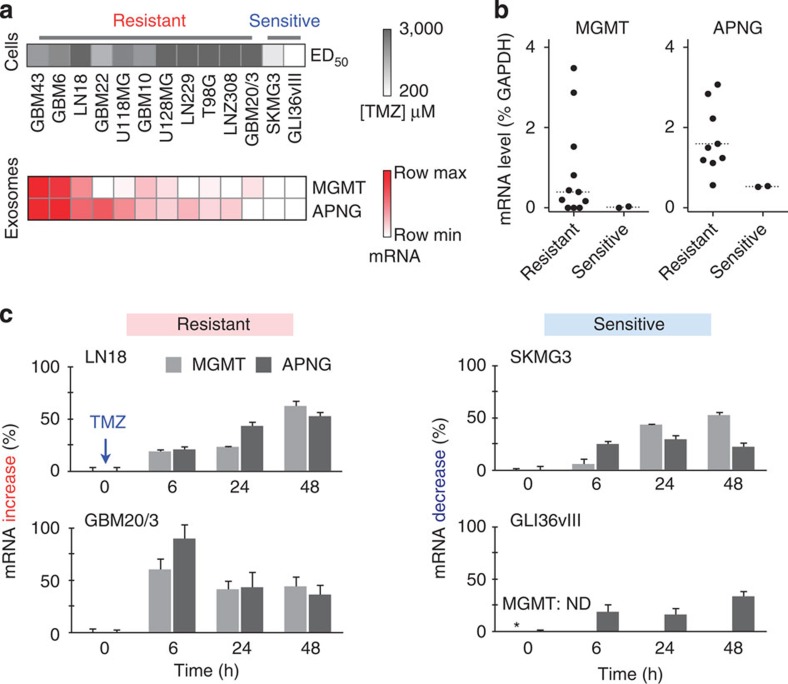
Effects of TMZ treatment. (**a**) Exosomal MGMT and APNG mRNA levels correlate with *in vitro* TMZ sensitivity (ED_50_). Cell lines were treated with varying doses of TMZ to determine their respective drug sensitivities (top panel). iMER analysis revealed that the levels of MGMT, APNG or both were elevated in resistant cell lines, whereas they were both low in sensitive ones (bottom panel). Row maximum and minimum refer, respectively, to the highest and lowest exosomal mRNA expression for a target marker across different GBM cell lines. (**b**) Higher average levels of exosomal MGMT/APNG were observed in resistant cell lines than in sensitive ones. However, there was overlap in exosomal mRNA levels between resistant and sensitive cell lines, demonstrating that a single marker was unable to distinguish drug resistance. Dotted line indicates the mean. (**c**) Serial analysis of exosomal MGMT and APNG mRNA levels following TMZ treatment. In resistant cell lines, exosomal MGMT/APNG mRNA levels increased within hours of drug treatment, while, in sensitive cell lines, the exosomal target levels decreased. GLI36vIII cell lines expressed non-detectable exosomal MGMT mRNA (* MGMT: ND). All experiments were performed in triplicate and the data are shown as mean±s.d.

We next tested whether exosomal mRNA analysis can be used to predict treatment effects. With TMZ as the model drug, we chose to analyse mRNA for MGMT^2^ and APNG^3^, key enzymes that repair drug-induced DNA damages. We first treated a panel of GBM cell lines with varying doses of TMZ. According to their dose-responses, the cell lines were broadly classified as TMZ resistant (GBM43, GBM6, LN18, GBM22, U118MG, GBM10, U128MG, LN229, T98G, LNZ308 and GBM20/3) or sensitive cells (GLI36vIII and SKMG3) (Fig. 4a, top and Supplementary Fig. 10). For single marker analysis, the average exosomal levels of MGMT or APNG mRNA were higher in resistant cell lines than in sensitive ones (Fig. 4b), although the differences were not statistically significant (*P*=0.34 for MGMT, *P*=0.11 for APNG; two-tailed *t*-test) due to a large overlap. Further breakdown, however, revealed combinatorial contributions to drug resistance: the levels of MGMT, APNG or both were elevated in resistant cell lines, whereas they were both negligible in sensitive ones (Fig. 4a, bottom).

We next examined the kinetics of MGMT/APNG mRNA changes following TMZ treatment. In TMZ-resistant cell lines, we observed fairly rapid temporal increases in exosomal mRNA levels of MGMT, APNG, or both (Fig. 4c, left). In sensitive cell lines, the mRNA levels decreased (Fig. 4c, right). Surprisingly, these changes occurred within several hours of drug treatment. Exosomal mRNA changes correlated well with cellular mRNA changes (Supplementary Fig. 11) and cellular promoter DNA methylation changes (Supplementary Fig. 12).

### Analysis of clinical samples

To test the utility of the iMER assay in measuring treatment response, we conducted a clinical feasibility study. We aimed at addressing two questions: (1) how do exosomal mRNA levels of diagnostic markers compare between GBM patients and 2) what are the changes of exosomal mRNA during the course of TMZ treatment.

**Figure 5:**
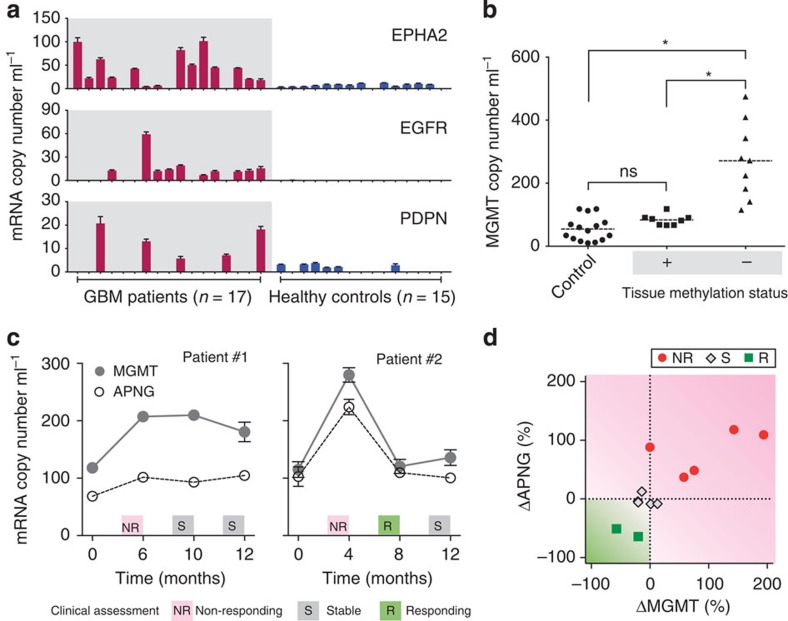
Analysis of clinical samples. (**a**) Measurement of mRNA for EPHA2, EGFR, PDPN in serum samples. GBM patients (*n*=17) generally showed higher levels of markers compared with healthy controls (*n*=15). (**b**) Correlation of exosomal MGMT mRNA against tumour tissue methylation status. Tissue methylation correlates inversely with exosomal MGMT copy number. The mRNA levels were significantly higher (*P<0.001; Tukey's multiple comparison test) in GBM patients with negative tissue MGMT methylation, than that of patients with positive methylation or healthy controls. The latter categories were non-distinguishable (NS) from each other. (**c**) Longitudinal exosomal MGMT and APNG mRNA analyses were performed in seven GBM patients; two representative examples are shown (see Supplementary Fig. 15 for other patients). Clinical assessments (NR, S, R) were based on radiological findings, clinical examination and lab values. All changes were plotted as mean±s.d. (**d**) Sequential exosomal mRNA changes between two time points were analysed in GBM patients (*n*=7) undergoing TMZ treatment. All changes were normalized to their initial values and plotted according to clinical evaluation at the end of the assessment period (the later time point). All changes were independent of initial tissue MGMT methylation status.

The exosomal levels of gene product diagnostic markers (EPHA2, EGFR and PDPN) were heterogeneous among clinical blood samples (Fig. 5a). No detectable EGFR mRNA was found in some GBM patients (*n*=7) despite the fact that we used EGFR protein for GBM enrichment (see Supplementary Fig. 13); such discrepancies between exosomal mRNA and protein levels have been observed in other tumour studies as well^14^. The average levels of EPHA2 and EGFR were significantly higher (*P*<0.05; two-tailed *t*-test) in GBM patients (*n*=17) than in healthy controls (*n*=15); PDPN level was similar in both groups (*P*=0.15; Supplementary Fig. 14a). Using a single marker alone, the iMER assay correctly identified GBM cases at the accuracies of 84.4% (EPHA2) and 78.1% (EGFR). The accuracy increased to 90% when the diagnosis was based on combined markers (Supplementary Fig. 14b).

We also measured exosomal MGMT mRNA in these clinical biofluids (Fig. 5b). The mRNA levels were found to be significantly higher (*P*<0.0001; Tukey's multiple comparison test) in GBM patients with negative MGMT promoter DNA methylation in primary tissues (see Methods for details on tissue analysis). Conversely, the levels were similar between healthy controls and methylation-positive GBM patients (*P*=0.61; Tukey's multiple comparison test). These analyses support the clinical use of the iMER assay as a non-invasive alternative to measuring promoter epigenetic methylation in glioblastoma tissue-derived genomic DNA.

Finally, we performed serial iMER analysis for MGMT and APNG in seven GBM patients undergoing treatment. Blind to the iMER assay results, treating physicians evaluated the patients' responses based on magnetic resonance imaging (MRI)^31^ and clinical neuro-oncologic metrics (for example, performance status, steroid dose changes and neurologic examination). Figure 5c and Supplementary Fig. 15 show longitudinal exosomal changes as related to clinical assessment to treatment. The initial levels of MGMT and APNG varied across patients, and neither was significantly correlated with the ultimate treatment outcome (Supplementary Fig. 16). In contrast, we observed qualitative match between exosomal mRNA changes and treatment trajectories (Fig. 5c and Supplementary Fig. 15). Further analysis (Fig. 5d) indicated that mRNA changes between two adjacent time points (ΔMGMT and ΔAPNG) could be used as predictor variables for treatment outcomes (multinomial logistic regression); we, however, note the need for larger cohort study to obtain statistical significance.

## Discussion

Analysis of extracellular vesicles is rapidly gaining recognition as an approach to diagnose, phenotype and prognosticate different cancers, such as GBM^1^. Although the presence of a few circulating tumour cells (CTCs) has been described in GBM patients^32^,^33^, exosome and CTC analyses are complementary. Tumour-derived exosomes have the advantages that they represent the heterogeneity of the tumour, are very abundant in blood, highly stable, readily pass the blood–brain barrier and can be analysed in small volumes (100 μl) of frozen serum/plasma samples. CTCs, in contrast, are relatively rare in blood and must be analysed in much larger volumes (7 ml) of fresh blood samples. In comparison with repeat GBM biopsies, exosomes can be assayed serially in serum and also report on the entirety of the tumour. We were thus particularly interested in harnessing the potential of exosomes to measure gene products relevant for therapy response. We show that this is feasible by developing a new exosomal RNA analysis platform, iMER, based on the enrichment of EGFR/EGFRvIII exosomes, and quantifying their mRNA contents in real time. The platform carries out magnetic separation, RNA extraction and RT-qPCR amplification in a single microfluidic chip. With the iMER platform, we demonstrated (i) exosomal mRNA levels correlated well with those of the cells of origin (13 mRNA targets × 14 cell lines, *R*^2^=0.918, Fig. 3a); (ii) MGMT and APNG mRNA can be reliably detected in patient biofluids; and (iii) serial measurements can be correlated to clinical response to temozolomide.

Previous research in our laboratory had focused on protein analysis of exosomes. For example, we have developed technologies to sensitively phenotype the protein contents of tumour exosomes by miniaturized nuclear magnetic resonance^12^ and by surface plasmon resonance^14^ methods and use these approaches for diagnostic purposes. On the basis of these protein measurements in additional patient cohorts (Supplementary Fig. 15) we developed the EGFR-based exosome enrichment method used in this study. However, what had remained more difficult was the analysis of intravesicular cargo such as nuclear proteins. While many nuclear targets play significant roles in conferring drug resistance, these proteins are variably partitioned into exosomes, presumably because of their primarily nuclear localization in cells. We thus argued that the mRNA counterparts of these nuclear targets, which are translated in the cytoplasm, could be more readily detected within exosomes. Here, we used an immunomagnetic bead approach to capture specific cancer exosomes before measurements. By optimizing the fluidic design and assay protocols of iMER, we achieved high yields both in exosome separation (>93%) and RNA extraction (150% as compared with commercial kit), thus accomplishing sensitive measurements of many mRNA targets for nuclear-localized proteins. For example, we showed that exosomal MGMT mRNA levels correlate with MGMT DNA promoter methylation status as a read-out of epigenetic silencing. The ability to monitor temozolomide resistance in biofluids is particularly important for adjusting treatments. In a specific patient example (patient #2 in Fig. 5c), TMZ resistance was circumvented by switching to another drug regimen (Bevacizumab/Avastin) to achieve better clinical outcomes.

The developed technology has a number of advantages but some challenges remain. Advantages include the simplicity, miniaturized on-chip processing (improving efficacy and eliminating sources of material loss), sensitivity, rapid turnaround time for analysis (∼2 h for complete analysis) and minimal sample volume requirement (∼100 μl of serum for multiplexed measurements). We envision further enhancing the technology. For example, the current PCR fluidics (four chambers) could be readily revised to accommodate more and smaller chambers; such a new design would enable higher-throughout and array-type multiplexed detection. Moreover, by using a cocktail of markers for immunomagnetic capture, the system could be expanded to enrich exosomes from highly heterogeneous and variable cancer subtypes. Combining iMER detection with exosomal protein analysis is another promising direction, which will provide more comprehensive snapshots of the tumour and improve mechanistic insights on potential translation of mRNA in recipient cells and regulation of protein translation. We have already developed fluidic-based platforms for exosomal protein analyses^12^,^14^; these platforms could be used to measure cancer-specific exosomes based on their protein markers, before target exosome lysis and iMER assays. Furthermore, in the current study we enriched for EGFR/EGFRvIII and used EGFR protein levels to identify tumour exosomes. However, for a general population of GBM patients, one could use other suitable markers for enhancing the detection of different tumour types. Ultimately, a panel, multiplexed approach will likely be the best strategy to sample from a variety of different GBM genetic backgrounds. Finally, the current study represents a feasibility study and requires future validation in larger cohorts.

The clinical implications of the developed technology are potentially broad. Efficient therapy assessment is still often a ‘black box' in the clinic due to a lack of sufficiently sensitive read-outs. Advanced imaging is certainly the current gold standard for brain tumours, but has also been shown to be a late-stage read-out and can be ambiguous in patients who had multiple operations, radiation therapy and are undergoing experimental treatments^1^. One of the key questions is how best to titrate temozolomide treatment for maximum efficiency and/or to choose alternative treatments in case of resistance. We believe that the described analysis of exosomal mRNA for MGMT and APNG represents one such approach. In addition, current measurements could be readily extended to include other resistance genes and/or read-outs of drug therapy in large cohorts in perspective trials. The resulting large data sets could then be critically analysed to validate the clinical utility of iMER. Finally, we originally developed the iMER technology for prognostic applications but it could be adapted for diagnostics, for example, to differentiate between different types of lesions detected on MRI scan (for example, brain abscess versus brain tumour). The predictive value of a positive exosome test could be quite high, if used in conjunction with an adjunctive test such as MRI. In this scenario, we conceive that patients with MRI scans and a clinical scenario highly suggestive of a brain tumour could receive a potentially confirmatory exosome test for genetic alterations consistent with tumour, which would obviate or reduce the need for biopsy in a population of glioblastoma patients. One could conceive that such diagnostic utility could also be extended to determine the genetic subtype of GBM, and hence a prediction of optimal treatment options and prognosis.

## Methods

### Cell culture

All human glioblastoma multiforme (GBM) cell lines were cultured in Dulbecco's modified essential medium (DMEM, Cellgro) containing 10% fetal bovine serum (FBS, Cellgro) and supplemented with penicillin-streptomycin (Cellgro). T98G, U138MG, LN229, LN18, U118 were obtained from American Type Culture Collection (ATCC). LNZ308 was provided by Dr Mikael Pittet, Massachusetts General Hospital (MGH). GLI36vIII (over-expressing human EGFRvIII) and GBM20/3 were generated from primary GBM samples^4^, SKMG3 was provided by Dr Timothy Chan, Memorial Sloan-Kettering Cancer Center. GBM6, GBM10, GBM22 and GBM43 were provided by Dr Alan Charest, Tufts University. Normal human astrocytes (NHA, Lonza) were cultured in astrocyte basal medium supplemented with SingleQuots, as recommended by the manufacturer. All cell lines were tested and free of mycoplasma contamination (MycoAlert Mycoplasma Detection Kit, Lonza, LT07–418).

### Exosome isolation

Cells at passage 1–15 were cultured in exosome-free medium (containing 5% exosome-depleted FBS) for 48 h. Conditioned medium from ∼10^7^ cells was collected, filtered through a 0.8-μm filter (Millipore) and concentrated (16,500 *g* for 20 min and 110,000 *g* for 70 min)^4^,^34^. For exosome isolation from clinical samples, human sera were obtained from venipuncture and used unpooled. Whole-blood samples were collected in non-citrated vacutainer tubes (Becton Dickinson) from both GBM patients and healthy donors under Institutional Review Board approved protocols and utilizing standard operating procedures. Sera were processed following clot formation for 30 min. After brief centrifugation (1,100 g for 10 min), sera were sterile-filtered through a 0.8-μm filter (Millipore) and frozen at –80 °C within 2 h of collection. Thawed samples were used unidentified and directly for target-specific exosome isolation as described below. For measurement of exosome size distribution and concentration, we used the nanoparticle tracking analysis (NTA) system (LM10, Nanosight). For optimal counting, exosome concentrations were adjusted to obtain ∼50 exosomes in the field of view and all NTA measurements were performed with identical experiment settings for consistency.

### Fabrication of microfluidic device

The microfluidic cartridge was fabricated by stacking two layers of polydimethylsiloxane (PDMS; Dow Corning) on a glass slide (25 mm × 75 mm). Cast moulds (50 μm thickness) were prepared by patterning epoxy-based SU8–3050 photoresist (Microchem) on silicon wafers via conventional photolithography. Microchannels and site-locators for torque-actuated valves were then replicated by pouring uncured PDMS to the cast molds (1 mm thickness). After curing the bottom PDMS layer, 8 bolt-nut pairs (#4–40) were aligned and glued on the bottom layer with uncured PDMS. Once the bolt-nut pairs were bonded to the layer, uncured PDMS polymer was poured over the assembly, forming the second layer (final thickness, 3 mm). After polymer curing, inlets, outlets and reservoirs were punched out. The maximum reservoir volume was 60 μl. The assembled PDMS pieces were bonded irreversibly to a glass slide.

### Device preparation

Before operating the microfluidic device for immunomagnetic capture and RNA extraction, silica beads (20 μm, Thermo Scientific) were introduced for subsequent RNA isolation. To confine and densely pack the beads within the main RNA extraction chamber, weir-shaped physical barriers^35^ were implemented at three boundaries around the chamber, with the exception of the bead inlet. The entire device was then flushed with cycles of RNaseZap (Life Technologies), RNase-free water (Life Technologies) and ethanol (Sigma), before being dried under vacuum.

### Immunomagnetic capture

Capturing antibodies were conjugated onto magnetic beads for target-specific exosome isolation. In brief, targeting antibodies against EGFR and EGFRvIII (Cetuximab, Bristol-Myers Squibb), CD63 (clone H5C6, BD Biosciences) or respective IgG isotype control antibodies (Biolegend) were biotinylated (Thermo Scientific) at room temperature for 1 h and buffer-exchanged using Zeba desalting columns (Thermo Scientific). Excess antibodies were then incubated with neutravidin-coated magnetic polystyrene particles (3 μm, Spherotech) at room temperature before repeated magnetic pull-down purification. Exosome-containing solutions, either concentrated from cell culture conditioned media or thawed sera, were then incubated with immunomagnetic particles for 15 min at room temperature.

### Enzyme-linked immunosorbant assay

Exosomes were adsorbed onto ELISA plates (Thermo Scientific) and blocked in PBS containing 1% bovine serum albumin (BSA, Sigma) for ELISA measurements. For determination of immunomagnetic capture efficiency, exosomes in the original mixture and post-capture supernatant were adsorbed as previously described. After washing, antibodies (1 μg ml^−1^) were added in blocking solution and incubated for 2 h at room temperature. Following incubation with horseradish peroxidase-conjugated secondary antibody (Thermo Scientific), chemiluminescence signals were determined (Safire, Tecan).

### Western blotting analysis

Exosomes were lysed in radio-immunoprecipitation assay buffer containing protease inhibitors (Thermo Scientific) and protein concentration quantified using the bicinchoninic acid assay (BCA assay, Thermo Scientific). Protein lysates were resolved by sodium dodecyl sulfate polyacrylamide gel electrophoresis (SDS–PAGE), transferred onto polyvinylidene fluoride membranes (PVDF, Invitrogen) and immunoblotted with antibodies against EGFR (D38B1, Cell Signaling), CD63 (sc-5275, Santa Cruz), MGMT (MT3.1, EMD/Millipore), APNG (sc-101237, Santa Cruz) and GAPDH (14C10, Cell Signaling). All antibodies were used at 1,000-fold dilution (Cell Signaling) or at a concentration of 1 μg ml^−1^ (Santa Cruz and EMD/Millipore). Following incubation with a horseradish peroxidase-conjugated secondary antibody (Cell Signaling), chemiluminescence was used for immunodetection (Thermo Scientific). Full images are presented in Supplementary Figs 4 and 8.

### Scanning electron microscopy

All samples were fixed with half-strength Karnovsky's fixative and washed twice with PBS. After dehydration in a series of increasing ethanol concentrations, samples were transferred for critical drying (Samdri, Tousimis) and subsequently coated with platinum/palladium using a sputter coater (208HR, Cressington Scientific Instruments), before imaging with a scanning electron microscope (Supra55VP, Carl Zeiss).

### RNA extraction and quantification

Captured exosomes were lysed in 40 μl of guanidine-containing buffer (Qiagen) with 70% ethanol (Sigma). For on-chip RNA extraction, the sample was flown through the RNA extraction chamber at 0.5 ml h^−1^, where RNA binds to densely packed silica beads (20 μm, Fisher Scientific). Subsequent flushing with 40 μl of wash buffer containing DNase I (Qiagen) and 40 μl of ethanol at 0.5 ml h^−1^ was used to remove traces of DNA and contaminants. Finally, RNA was eluted in 10 μl of DEPC-treated water (Life Technologies). For comparison of RNA extraction yield and quality, aliquots of the same exosome lysate were processed, respectively, by the iMER platform and a commercially available kit (RNeasy Micro, Qiagen), per manufacturer's protocol. Extracted RNA samples were quantified with Nanodrop spectrophotometer (Thermo Scientific) and evaluated with 2100 Bioanalyzer (Agilent) using a RNA 6000 Pico Chip.

### mRNA analysis

Extracted RNA was reverse-transcribed to generate first-strand cDNA (High-Capacity cDNA Reverse Transcription, Applied Biosystems and QuantiTect Reverse Transcription, Qiagen) and pre-amplified when necessary (Taqman PreAmp Master Mix, Life Technologies) before qPCR. All reactions were performed using Taqman Fast Advanced Master Mix (Life Technologies) and 100 nM of each primer set (Taqman gene expression assays, Life Technologies) as recommended by the manufacturer. Amplification conditions consisted of 1 cycle of 95 °C for 30 s, 40 cycles of 95 °C for 3 s and 60 °C for 30 s. For mRNA analysis with conventional RT-qPCR, cycling was performed on an ABI 7500 Fast Real-Time PCR system (Applied Biosystems). For on-chip analysis with the iMER platform, reaction mixtures were sealed with 2 μl mineral oil (Sigma) to prevent evaporation during thermal cycling. At the end of each amplification cycle, target signal was detected via a miniaturized fluorescence meter (ESElog, Qiagen). All experiments were done in triplicate and relative quantification was done for each sample by normalizing with respective GAPDH expression.

### Clinical samples

GBM patients and healthy volunteers were recruited according to an Institutional Review Board approved protocol with informed consent. A total of 32 individuals were enroled. For the profiling study, we obtained serum samples from cancer patients (*n*=17), harbouring newly diagnosed or recurrent glioblastoma, as well as healthy volunteers (*n*=15). Cancer diagnoses were confirmed by neuropathologic examination and clinical imaging. Methylation status of MGMT in cancer patients were determined on pathologic tissues using methylation-specific PCR, performed independently by the Massachusetts General Hospital Pathology Service. For longitudinal evaluation, serial serum samples were collected from each patient (*n*=7) at distinctive treatment follow-ups. All serum samples were filtered through a 0.8-μm filter (Millipore) to remove cells and debris and used directly for immunomagnetic capture before mRNA analyses. All patients received standard-of-care TMZ treatment. Responder, stable and non-responder status were assigned by a neuro-oncologist (F.H.H.), without knowledge of iMER results, based on commonly used response criteria: (1) MRI evaluation for presence of gadolinium enhancement changes based on bi-dimensional accepted criteria^31^ and (2) clinical assessment (for example, performance status, steroid dose changes and neurologic examination).

### Promoter methylation sequencing

Genomic DNA was extracted from GBM cell culture after cell lysis and proteinase K digestion (Qiagen). Purified DNA was quantified with Nanodrop spectrophotometer (Thermo Scientific). One microgram of genomic DNA was subjected to bisulfite conversion with the EpiTect bisulfite kit (Qiagen) according to the manufacturer's instructions, where cytosines are converted to uracil while 5-methylcytosines remain inert. For MGMT promoter amplification, the following primers were used: MGMT-Bis forward (5′-GGATATGTTGGGATAGTT-3′), and MGMT-Bis reverse (5′-AAACTAAACAACACCTAAA-3′). PCR was performed with initial denaturation, followed by 38 cycles of 94 °C for 1 min, 47.5 °C for 1 min and 72 °C for 1 min. For APNG promoter amplification, the following primers were used: APNG-Bis forward (5′-GTTGGGAAGAGGATTTATTTTTA-3′), and APNG-Bis reverse (5′-TTAACCTATACTCTAACTCTACCCC-3′). The initial denaturation was followed by 38 cycles of 94 °C for 1 min, 52 °C for 1 min and 72 °C for 1 min. PCR products were resolved on a 1.8% agarose gel and specific bands were excised with a clean razor blade and purified with the QIAquick gel extraction system (Qiagen). Isolated PCR products were cloned using the TOPO TA cloning system (Invitrogen) and at least 12 clones per target sample were sequenced with Taq DyeDeoxy Terminator cycle sequencing (Applied Biosystems). After cycle sequencing and clean up, the DNA samples were resolved by capillary electrophoresis on an ABI 3730XL DNA analyser to translate the fluorescence signals into their corresponding base pair sequence for promoter methylation analysis. The extent of methylation was determined by calculating the percentage of unconverted cytosine (methylated-C) for specific CpG islands in the DNA promoter sequence.

### *In vitro* drug treatment

TMZ (Temodar, Schering Plough) was used to investigate the effects of GBM drug treatment. To determine the effective dose (ED_50_) of the drug, cells were seeded at a density of 2,000 cells per well in a 96-well plate overnight, and treated with the drug (TMZ) or vehicle (final concentration of 0.1% DMSO) for 4 days. Cell viability was assessed using the 3-(4,5-dimethylthiazol-2-yl)-5-(3-carboxymethoxyphenyl)-2-(4-sulfophenyl)-2H-tetrazolium inner salt (MTS) cell proliferation assay (Promega). For cellular and exosome analyses of drug effects, GBM cells were given a dosage of TMZ below their respective ED_50_ concentrations (∼10% ED_50_) before subsequent promoter methylation sequencing and mRNA analyses.

### Mouse model

All animal procedures were performed according to guidelines issued by the Committee of Animal Care of Massachusetts General Hospital. Human GBM cells GLI36vIII and GBM10, determined to be methylated and unmethylated at MGMT promoter with bisulfite sequencing, as described above, were implanted (1 × 10^6^ cells) into the flanks of 6-week-old, female immunodeficient *nu/nu* mice (Charles River Laboratories). Xenograft tumours were allowed to grow for 2 weeks before animal sacrifice. Sera were collected via terminal cardiac puncture and processed for exosome analysis, as described above.

### Immunohistochemistry

Tumour implants were excised from euthanized mice and flash-frozen in a chilled isopentane bath on dry ice. Frozen sections (6 μm) were prepared and stored at −80 °C until staining was performed. Sections were blocked using 4% normal goat serum in PBS for 30 min before incubation with an anti-MGMT antibody (EPR4397, Abcam) at 4 °C overnight. The following day, sections were incubated with biotinylated anti-rabbit IgG (BA1000, Vector Laboratories) for 30 min at room temperature. VECTASTAIN ABC kit (Vector Laboratories) and AEC substrate (Dako) were used for colour development. All sections were counterstained with Harris hematoxylin (Sigma) and slides were scanned using a digital scanner (Nanozoomer 2.0RS, Hamamatsu).

### Statistical analysis

All measurements were performed in triplicate and the data are displayed as mean±s.d. For clinical samples, the measured mRNA levels (EPHA2, EGFR and PDPN) were analysed via logistic regression, using the disease state as a dichotomous outcome. A classification table that summarized the validity of predicted probabilities (Supplementary Fig. 14b) was constructed using standard formulas for diagnostic metrics (that is, sensitivity, specificity, accuracy), and receiver operation characteristic (ROC) curves were generated from the predicted probabilities. We used the R package (version 3.1.1) for ROC curve analyses. In evaluating the measured exosomal mRNA levels against tissue methylation in clinical samples, Tukey's multiple comparison test was employed and a *P*<0.05 was considered statistically significant. All other comparisons were performed with unpaired two-tailed *t*-test, and *P*<0.05 was considered statistically significant.

## Author contributions

H.S., J.C., H.L. and R.W. designed the study, analysed data, prepared figures and wrote the manuscript. H.S., J.C., K.L., L.B. and C.M. performed the research. B.S.C. and F.H.H. provided clinical samples. L.B. and X.O.B. generated cell lines and assisted in designing experiments. All authors contributed to the manuscript. R.W. and X.O.B. provided funding.

## Additional information

**How to cite this article:** Shao, H. *et al*. Chip-based analysis of exosomal mRNA mediating drug resistance in glioblastoma. *Nat. Commun.* 6:6999 doi: 10.1038/ncomms7999 (2015).

## Supplementary Material

Supplementary Information Supplementary Figures 1-16.

## References

[b1] Hochberg F. H. . Glioma diagnostics and biomarkers: an ongoing challenge in the field of medicine and science. Expert Rev. Mol. Diagn. 14, 439–452 (2014). 24746164 10.1586/14737159.2014.905202 PMC5451266

[b2] Hegi M. E. . MGMT gene silencing and benefit from temozolomide in glioblastoma. N. Engl. J. Med. 352, 997–1003 (2005). 15758010 10.1056/NEJMoa043331

[b3] Agnihotri S. . Alkylpurine-DNA-N-glycosylase confers resistance to temozolomide in xenograft models of glioblastoma multiforme and is associated with poor survival in patients. J. Clin. Invest. 122, 253–266 (2012). 22156195 10.1172/JCI59334 PMC3248301

[b4] Skog J. . Glioblastoma microvesicles transport RNA and proteins that promote tumour growth and provide diagnostic biomarkers. Nat. Cell Biol. 10, 1470–1476 (2008). 19011622 10.1038/ncb1800 PMC3423894

[b5] Simpson R. J., Lim J. W., Moritz R. L. & Mathivanan S. Exosomes: proteomic insights and diagnostic potential. Expert Rev. Proteomics 6, 267–283 (2009). 19489699 10.1586/epr.09.17

[b6] Taylor D. D., Zacharias W. & Gercel-Taylor C. Exosome isolation for proteomic analyses and RNA profiling. Methods Mol. Biol. 728, 235–246 (2011). 21468952 10.1007/978-1-61779-068-3_15

[b7] Thery C., Zitvogel L. & Amigorena S. Exosomes: composition, biogenesis and function. Nat. Rev. Immunol. 2, 569–579 (2002). 12154376 10.1038/nri855

[b8] Lotvall J. . Minimal experimental requirements for definition of extracellular vesicles and their functions: a position statement from the International Society for Extracellular Vesicles. J. Extracell. Vesicles 3, 26913 (2014). 25536934 10.3402/jev.v3.26913 PMC4275645

[b9] Thery C., Ostrowski M. & Segura E. Membrane vesicles as conveyors of immune responses. Nat. Rev. Immunol. 9, 581–593 (2009). 19498381 10.1038/nri2567

[b10] Mathivanan S., Ji H. & Simpson R. J. Exosomes: extracellular organelles important in intercellular communication. J. Proteomics 73, 1907–1920 (2010). 20601276 10.1016/j.jprot.2010.06.006

[b11] Graner M. W. . Proteomic and immunologic analyses of brain tumor exosomes. FASEB J. 23, 1541–1557 (2009). 19109410 10.1096/fj.08-122184 PMC2669426

[b12] Shao H. . Protein typing of circulating microvesicles allows real-time monitoring of glioblastoma therapy. Nat. Med. 18, 1835–1840 (2012). 23142818 10.1038/nm.2994 PMC3518564

[b13] Chen W. W. . BEAMing and droplet digital PCR analysis of mutant IDH1 mRNA in glioma patient serum and cerebrospinal fluid extracellular vesicles. Mol. Ther. Nucleic Acids 2, e109 (2013). 23881452 10.1038/mtna.2013.28 PMC3732870

[b14] Im H. . Label-free detection and molecular profiling of exosomes with a nano-plasmonic sensor. Nat. Biotechnol. 32, 490–495 (2014). 24752081 10.1038/nbt.2886 PMC4356947

[b15] Bredel M. & Zentner J. Brain-tumour drug resistance: the bare essentials. Lancet. Oncol. 3, 397–406 (2002). 12142169 10.1016/s1470-2045(02)00786-6

[b16] Madhusudan S. & Middleton M. R. The emerging role of DNA repair proteins as predictive, prognostic and therapeutic targets in cancer. Cancer Treat. Rev. 31, 603–617 (2005). 16298073 10.1016/j.ctrv.2005.09.006

[b17] Weibel D. B. . Torque-actuated valves for microfluidics. Anal. Chem. 77, 4726–4733 (2005). 16053282 10.1021/ac048303p

[b18] Rho J. . Magnetic nanosensor for detection and profiling of erythrocyte-derived microvesicles. ACS Nano 7, 11227–11233 (2013). 24295203 10.1021/nn405016y PMC3898036

[b19] Mishima K. . Increased expression of podoplanin in malignant astrocytic tumors as a novel molecular marker of malignant progression. Acta Neuropathol. 111, 483–488 (2006). 16596424 10.1007/s00401-006-0063-y

[b20] Wykosky J., Gibo D. M., Stanton C. & Debinski W. EphA2 as a novel molecular marker and target in glioblastoma multiforme. Mol. Cancer Res. 3, 541–551 (2005). 16254188 10.1158/1541-7786.MCR-05-0056

[b21] Townsend D. M. & Tew K. D. The role of glutathione-S-transferase in anti-cancer drug resistance. Oncogene 22, 7369–7375 (2003). 14576844 10.1038/sj.onc.1206940 PMC6361125

[b22] Chen H. Y., Shao C. J., Chen F. R., Kwan A. L. & Chen Z. P. Role of ERCC1 promoter hypermethylation in drug resistance to cisplatin in human gliomas. Int. J. Cancer 126, 1944–1954 (2010). 19626585 10.1002/ijc.24772

[b23] Galluzzi L. . Molecular mechanisms of cisplatin resistance. Oncogene 31, 1869–1883 (2012). 21892204 10.1038/onc.2011.384

[b24] Wrensch M. . ERCC1 and ERCC2 polymorphisms and adult glioma. Neuro Oncol. 7, 495–507 (2005). 16212814 10.1215/S1152851705000037 PMC1871723

[b25] Berger W. . Overexpression of the human major vault protein in astrocytic brain tumor cells. Int. J. Cancer 94, 377–382 (2001). 11745417 10.1002/ijc.1486

[b26] Steiner E., Holzmann K., Elbling L., Micksche M. & Berger W. Cellular functions of vaults and their involvement in multidrug resistance. Curr. Drug Targets 7, 923–934 (2006). 16918321 10.2174/138945006778019345

[b27] Kuan C. T. . MRP3: a molecular target for human glioblastoma multiforme immunotherapy. BMC Cancer 10, 468 (2010). 20809959 10.1186/1471-2407-10-468 PMC2940806

[b28] Qi L. . Heterogeneity of primary glioblastoma cells in the expression of caspase-8 and the response to TRAIL-induced apoptosis. Apoptosis 16, 1150–1164 (2011). 21877214 10.1007/s10495-011-0645-6 PMC3257579

[b29] Mehrian-Shai R. . Insulin growth factor-binding protein 2 is a candidate biomarker for PTEN status and PI3K/Akt pathway activation in glioblastoma and prostate cancer. Proc. Natl Acad. Sci. USA 104, 5563–5568 (2007). 17372210 10.1073/pnas.0609139104 PMC1838515

[b30] Pols M. S. & Klumperman J. Trafficking and function of the tetraspanin CD63. Exp. Cell Res. 315, 1584–1592 (2009). 18930046 10.1016/j.yexcr.2008.09.020

[b31] Wen P. Y. . Updated response assessment criteria for high-grade gliomas: response assessment in neuro-oncology working group. J. Clin. Oncol. 28, 1963–1972 (2010). 20231676 10.1200/JCO.2009.26.3541

[b32] Muller C. . Hematogenous dissemination of glioblastoma multiforme. Sci. Transl. Med. 6, 247ra101 (2014). 10.1126/scitranslmed.3009095 25080476

[b33] Sullivan J. P. . Brain tumor cells in circulation are enriched for mesenchymal gene expression. Cancer Discov. 4, 1299–1309 (2014). 25139148 10.1158/2159-8290.CD-14-0471 PMC4221467

[b34] Balaj L. . Tumour microvesicles contain retrotransposon elements and amplified oncogene sequences. Nat. Commun. 2, 180 (2011). 21285958 10.1038/ncomms1180 PMC3040683

[b35] Chung J. . Microfluidic cell sorter (muFCS) for on-chip capture and analysis of single cells. Adv. Healthcare Mater. 1, 432–436 (2012). 10.1002/adhm.201200046 PMC3508764 23184773

