# Supplementary material for: Chip-based analysis of exosomal mRNA mediating drug resistance in glioblastoma
Source: Nat Commun. 2015 May 11;6:6999. doi: 10.1038/ncomms7999 (PMC4430127; doi:10.1038/ncomms7999)
Supplement: Supplementary Information — Supplementary Figures 1-16. [file ncomms7999-s1.pdf]

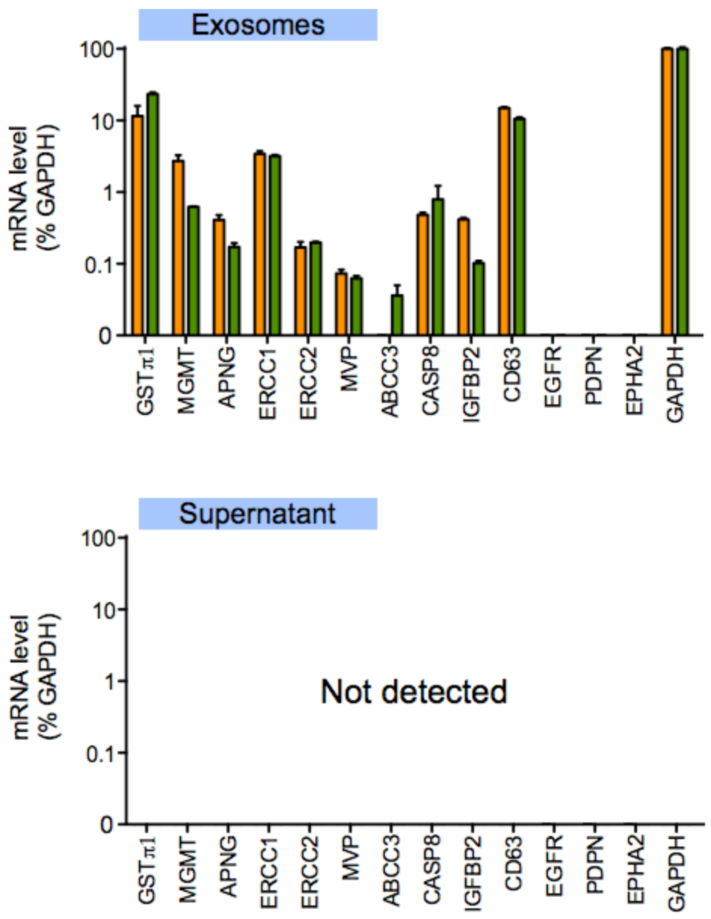

Supplementary Figure 1. mRNA targets were found in exosomes and absent in free-floating supernatant. Serum exosomes and exosome-free supernatant were separated via ultracentrifugation and lysed to analyze their mRNA contents. Note that mRNA markers were found in exosomes but absent in supernatant. Sera from healthy volunteers were used. All measurements were performed in triplicate and the data are shown as mean ± s.d.

a.

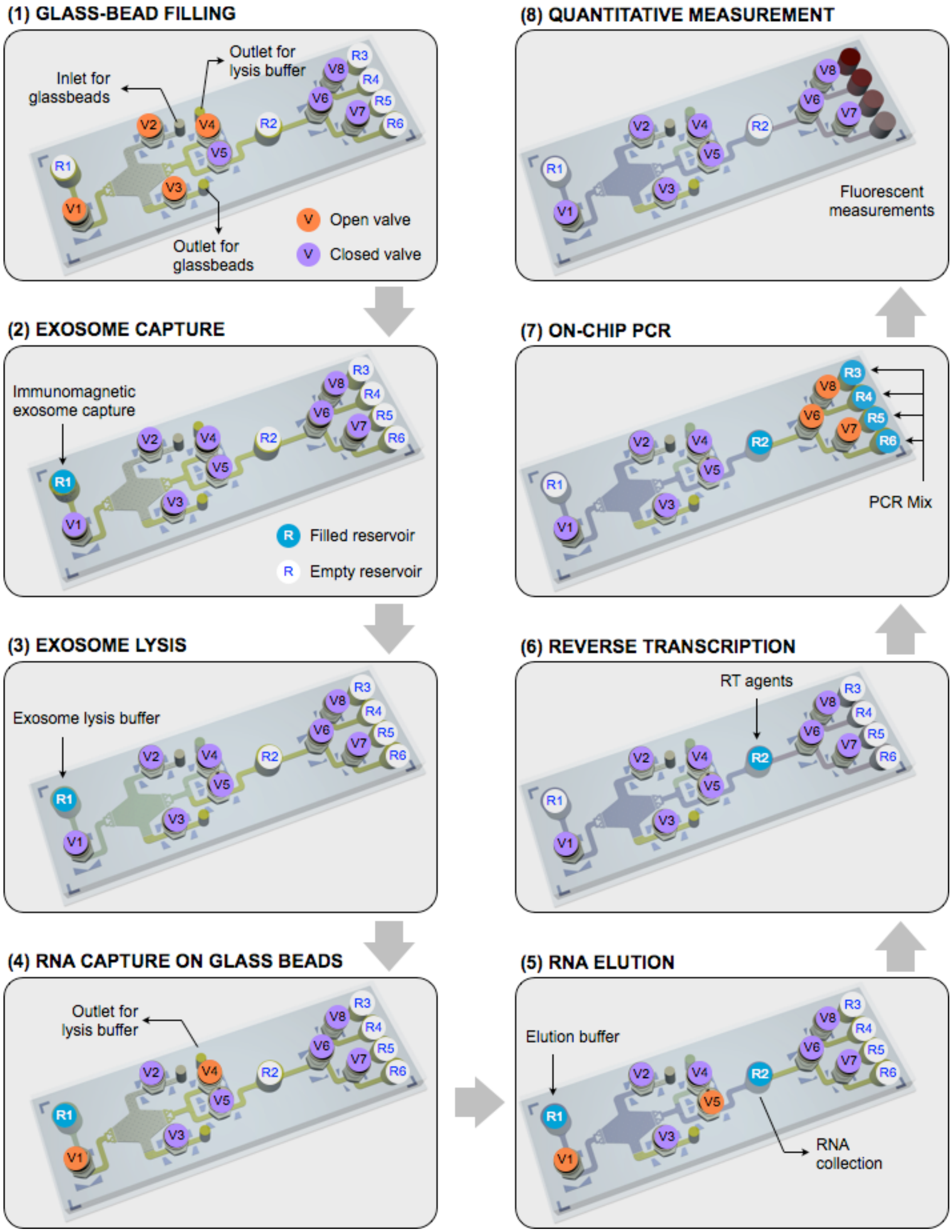
(1) GLASS-BEAD FILLING
Inlet for glassbeads
Outlet for lysis buffer
Outlet for glassbeads
Open valve
Closed valve
(2) EXOSOME CAPTURE
Immunomagnetic exosome capture
Filled reservoir
Empty reservoir
(3) EXOSOME LYSIS
Exosome lysis buffer
(4) RNA CAPTURE ON GLASS BEADS
Outlet for lysis buffer
(5) RNA ELUTION
Elution buffer
RNA collection
(6) REVERSE TRANSCRIPTION
RT agents
(7) ON-CHIP PCR
PCR Mix
(8) QUANTITATIVE MEASUREMENT
Fluorescent measurements

b.

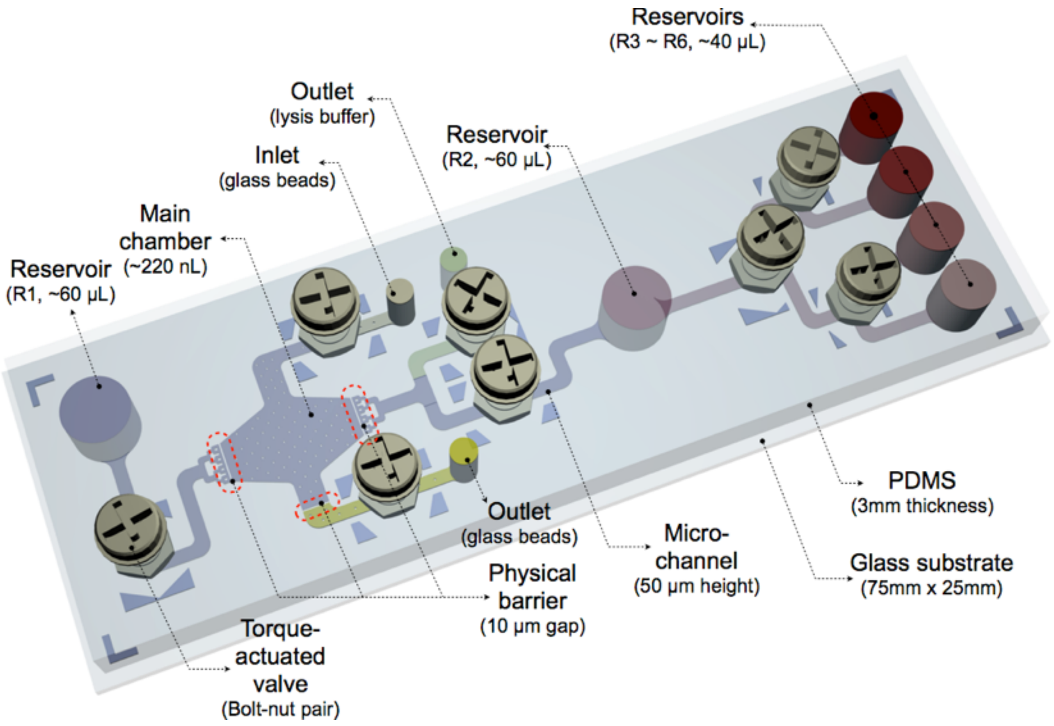

Supplementary Figure 2. The iMER fluidic chip. (a) Device operation. (i) Glass beads (20 μm in diameter) suspended in ethanol (100%) are introduced into the main chamber through the inlet while torque-actuated valves, V1~V4, are open. The beads are collected only in the chamber due to weir-style physical barriers fabricated around the chamber. Excess alcohol is collected and removed from reservoir (R1), outlets for glass beads and lysis buffer. (ii) A mixture of exosomes and immunomagnetic microbeads (3 μm) is loaded into R1 with V1 closed by screwing the bolt in. After a 15-minute incubation, a permanent magnet is placed under R1 to pull down the magnetic microbeads. Unbound exosomes are aspirated out. (iii) Lysis buffer (40 μL of guanidine-containing buffer, Qiagen) is added to R1. (iv) Exosome lysate flows through the glass-bead chamber for RNA capture. With V1 and V4 open, the lysate from R1 is driven into the chamber by negative pressure applied at the outlet for lysis buffer. The chamber is then sequentially flushed with 40 μL of wash buffer containing DNase 1 (Qiagen) and 40 μL 80% ethanol (Sigma). (v) Pure water is injected to elute the captured RNA from the beads. Negative pressure is applied at R2 to generate a fluidic flow from R1 to R2. (vi) Reverse transcription (RT) agents (QuantiTect Reverse Transcription, Qiagen) are added to R2, and the chip is heated to 42 °C. (vii) Transcribed DNA is diluted and distributed to four chambers by opening V6 ~ V8. Fluid flow was driven by gravitational energy difference in reservoirs. After distribution to four chambers, the microfluidic chip is placed on a custom-designed thermo-cycler for real-time PCR. Amplification conditions consisted of 1 cycle of 95 °C for 30 sec, 40 cycles of 95 °C for 3 sec and 60 °C for 30 sec. To prevent evaporation from the reservoirs during thermal cycling, we sealed the reaction mixtures with 2 μL mineral oil (Sigma). (viii) Fluorescence intensities are measured at each PCR cycle by a portable fluorescence meter (ESElog, Qiagen). **(b)** Key device dimensions. The maximum volume of each chamber is shown.

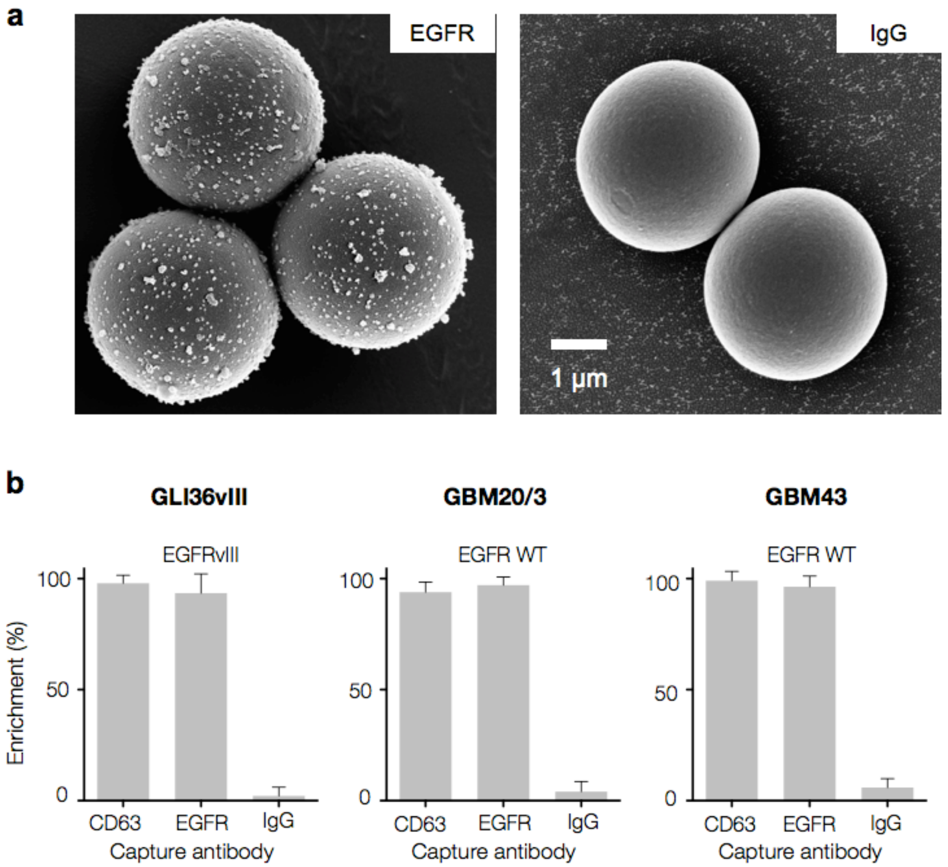

Supplementary Figure 3. Specificity of exosome capture with antibody-coated magnetic beads. (a) Immunomagnetic beads functionalized with anti-EGFR (left) and IgG control (right) antibodies respectively were incubated with equal amounts of exosomes derived from GLI36vIII human GBM culture. In agreement with quantitative analysis of exosome capture efficiency (**Fig. 2a** in the main text), scanning electron micrographs of the immunomagnetic beads after incubation confirmed highly specific exosomal enrichment with the anti-EGFR beads, whereas the control beads demonstrated negligible binding of exosomes. **(b)** Exosome enrichment efficiencies. Samples containing exosomes from GLI36vIII and primary GBM cell lines (GBM20/3 and GBM43) were incubated with magnetic immunobeads for exosome capture, and the respective enrichment efficiencies were determined.

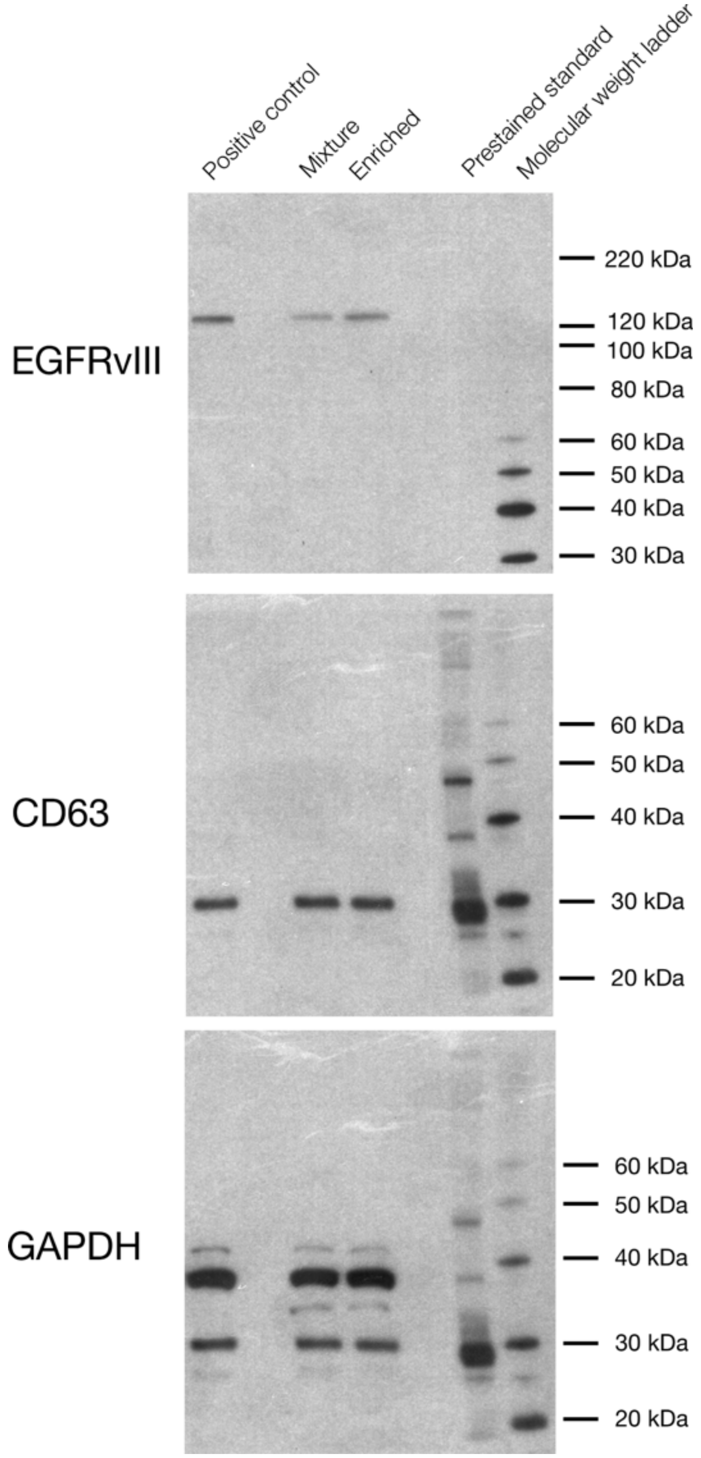

Supplementary Figure 4. Full Western blotting images for Figure 2b.

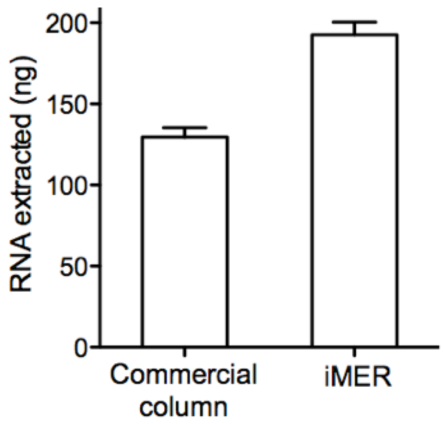

Supplementary Figure 5. Comparison of RNA extraction yields by iMER and commercial column. Equal volume (100 μL) of identical exosome lysates, isolated from GLI36vIII GBM cell culture were flown through the iMER platform and a commercial column (Qiagen) for total RNA extraction. RNA amounts quantified by a spectrophotometer indicated a higher extraction yield by the iMER platform. All measurements were performed in triplicate and the data are shown as mean ± s.d.

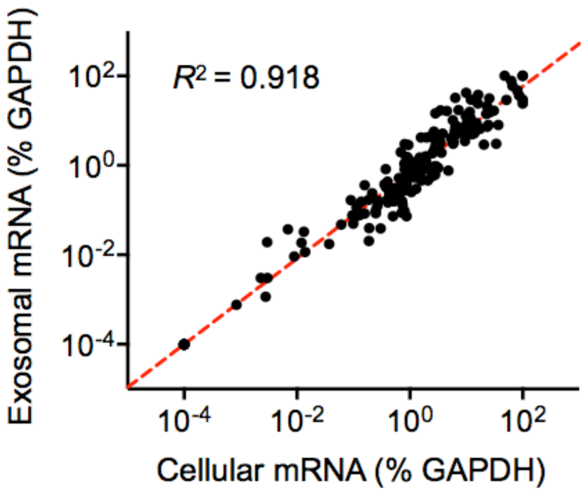

Supplementary Figure 6. Correlation between exosomal and cellular mRNA expressions. Expressions of mRNA transcripts (13 mRNA targets × 14 cell lines) were measured in exosomes (by iMER assay) and in their parent cells (by conventional qRT-PCR). Relative mRNA expression levels, normalized against respective GAPDH levels, displayed a good correlation between exosomes and cells. All measurements were performed in triplicate and the data are shown as mean.

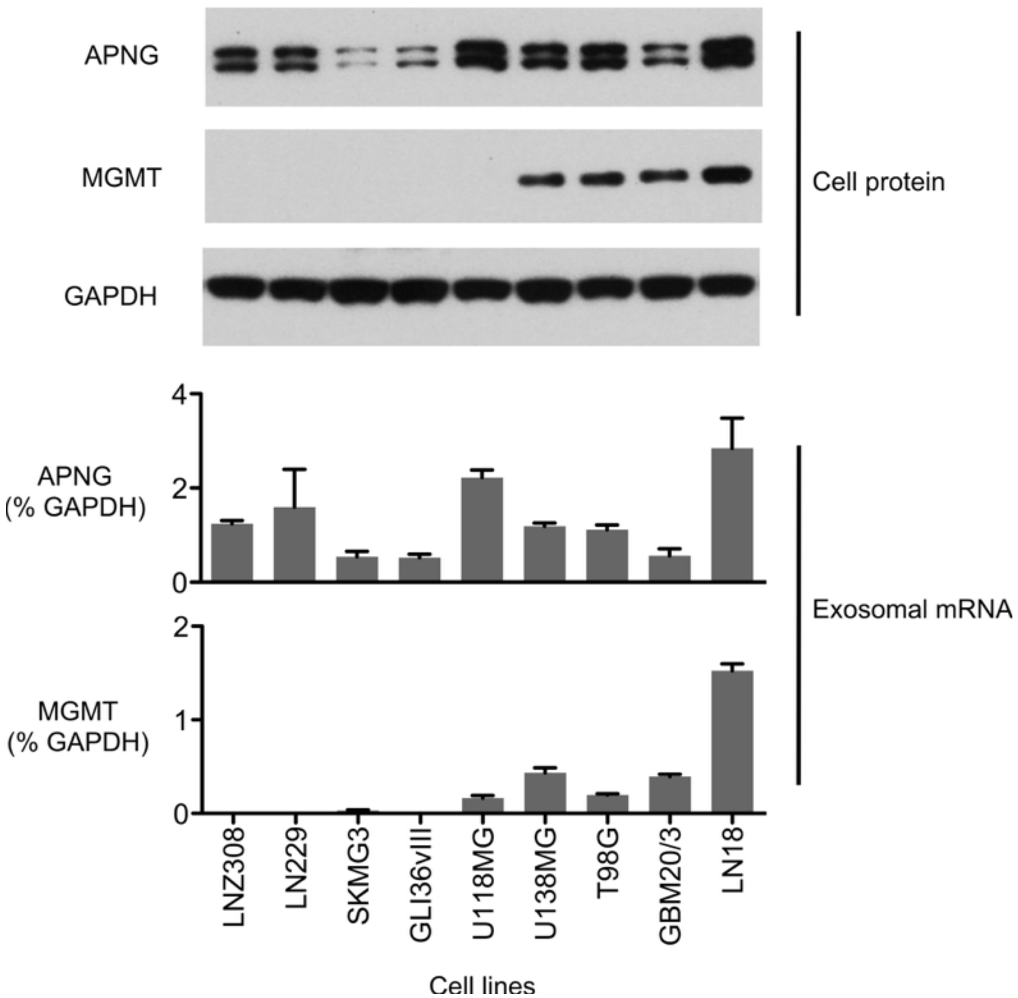

Supplementary Figure 7. Correlation of cellular protein expression with exosome mRNA levels. Cell lysates were immunoblotted to determine the cellular protein expression of APNG and MGMT. Exosomal mRNA levels correlated with cellular protein expressions. All measurements were performed with GAPDH as an intrinsic control. mRNA measurements were performed in triplicate and the data are shown as mean ± s.d.

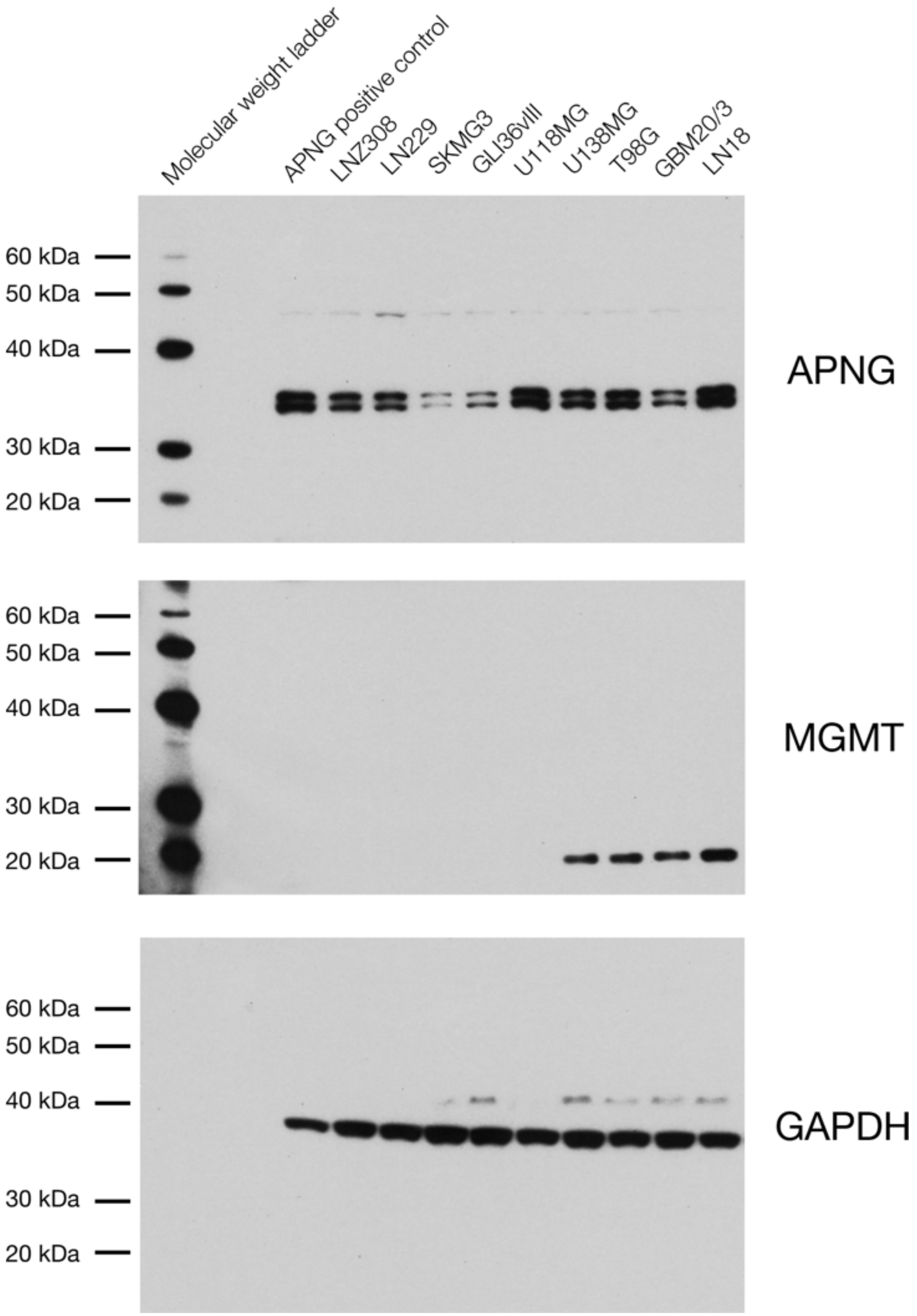

Supplementary Figure 8. Full Western blotting images for Supplementary Figure 7.

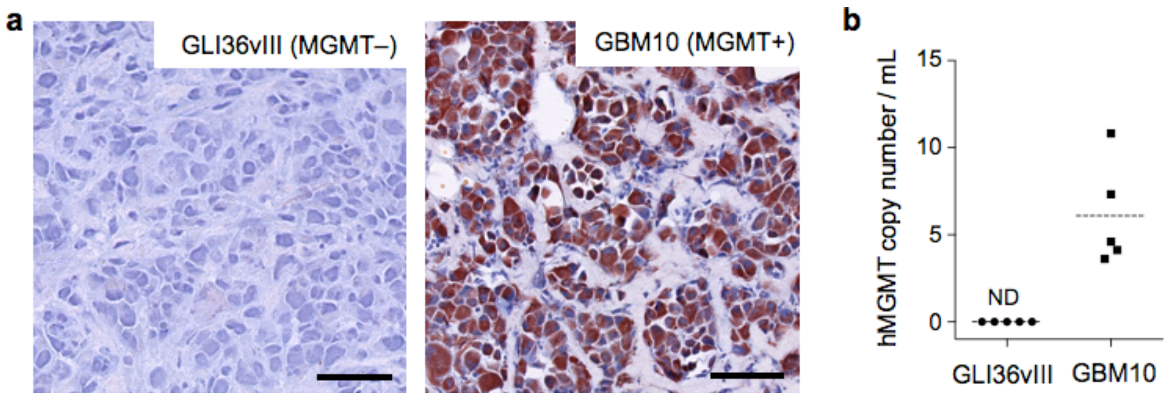

Supplementary Figure 9. iMER analysis in mouse xenograft models. Human GBM cells GLI36vIII (expressing negligible MGMT) and GBM10 (expressing high levels of MGMT) were implanted into the flanks of mice (*n* = 5 for each cell line). **(a)** Immunohistochemistry on extracted tumor tissues confirmed the different levels of MGMT protein expression, in agreement with *in vitro* mRNA analysis (**Fig. 3** in the main text). Scale bars indicate 100 μm. **(b)** Mouse serum samples were processed via the iMER platform to quantify human MGMT mRNA transcript (hMGMT). The exosome analysis distinguished between the two animal groups; the transcript was non-detectable (ND) in animals implanted with GLI36vIII, whereas exosomes from GBM10-implanted mice showed high levels of hMGMT mRNA.

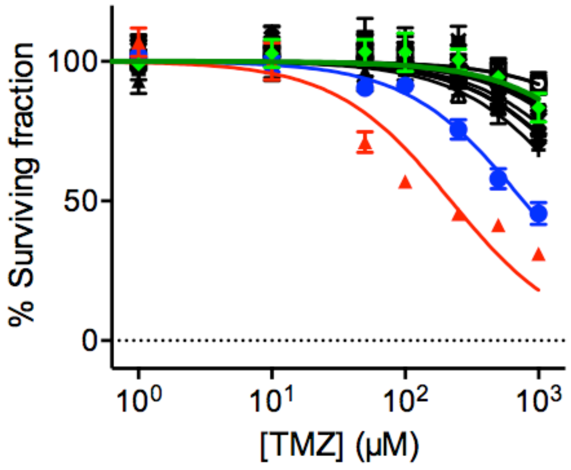

Cell line	ED50 (µM)
▲ GLI36vIII	220
● SKMG3	769
+ GBM22	2111
△ GBM6	2779
◆ GBM43	2796
▲ GBM10	2871
◇ U118MG	3437
▽ LN18	4851
▼ U138MG	5166
■ GBM20/3	5729
× LN229	5734
○ LNZ308	11207
□ T98G	11287
◆ NHA	6443

Supplementary Figure 10. Dose response of GBM cells when treated with TMZ. Human GBM cells were treated with varying doses of TMZ, and their viability was determined. Cell lines were classified into sensitive (GLI36vIII and SKMG3; ED_{50} < 1000 μM) and resistant (all other cell lines) according to their respective drug response. ED_{50}, 50% effective dose.

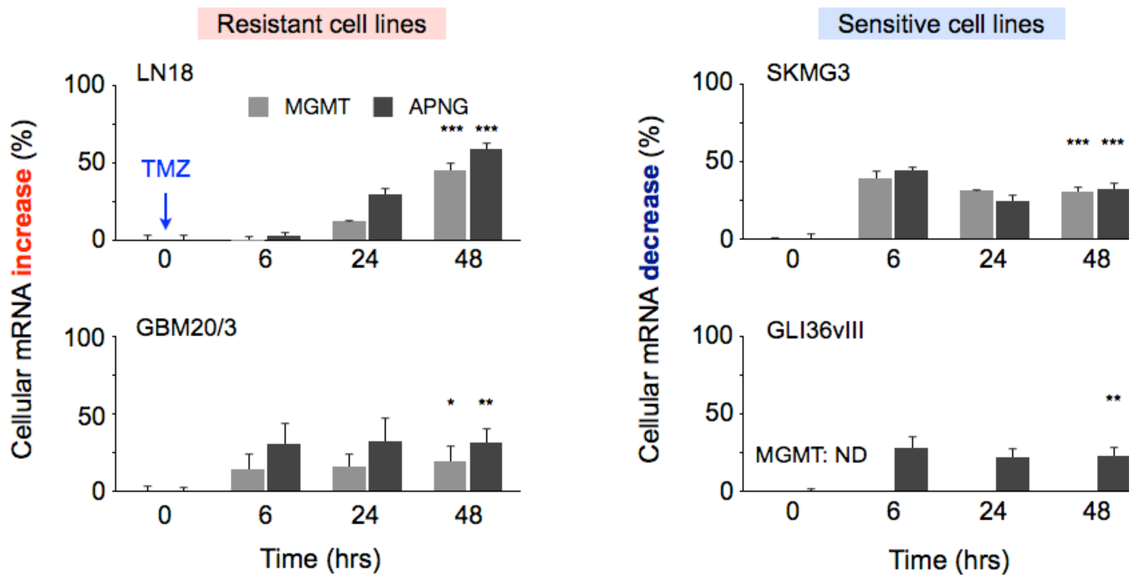

Supplementary Figure 11. Longitudinal analysis of MGMT and APNG mRNA levels in cells. Following TMZ treatment (at time = 0; blue arrow), cellular levels of MGMT/APNG mRNA were measured over time and plotted as relative changes against the initial (before the treatments). In resistant cell lines (**left**), cellular MGMT/APNG levels increased within hours of TMZ treatment, while in sensitive cell lines (**right**), the levels decreased. GLI36vIII cells expressed non-detectable (ND) cellular MGMT throughout the treatment course. All measurements were performed in triplicate and the data are shown as mean ± s.d. The initial (time = 0) and the final (time = 48 hrs) mRNA levels were compared with a two-tailed *t*-test (* $P < 0.05$, ** $P < 0.005$, *** $P < 0.0005$).

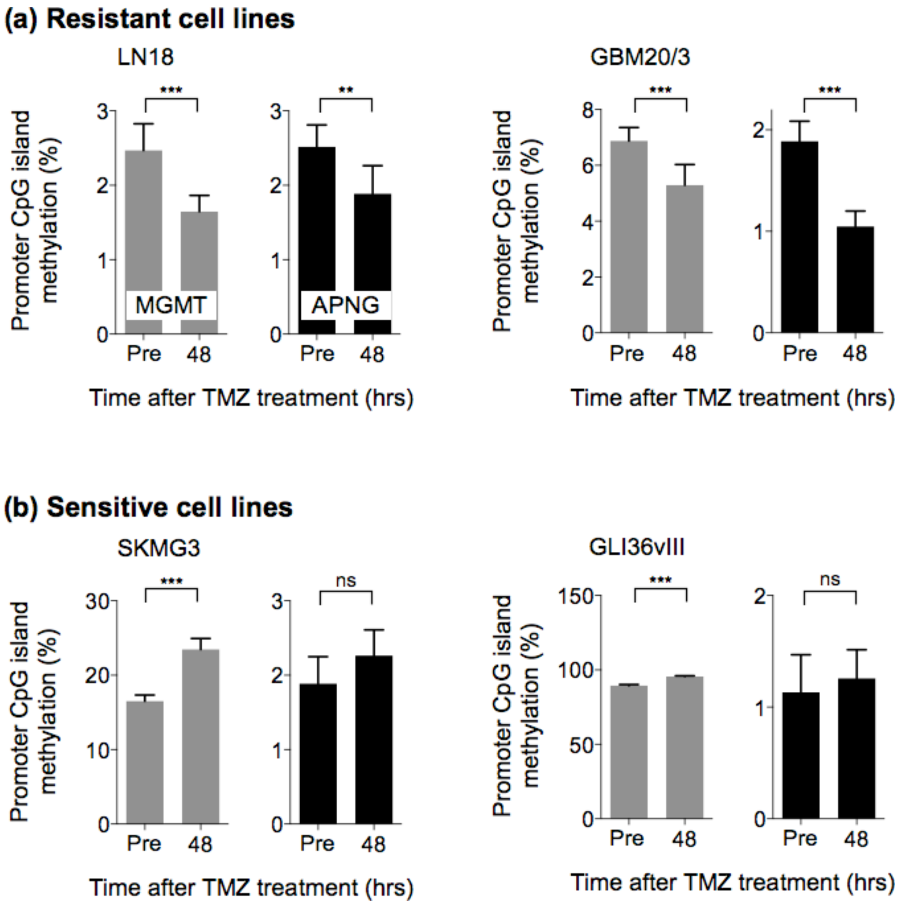

Supplementary Figure 12. Epigenetic methylation changes in DNA promoters of MGMT and APNG in cells following TMZ treatment. DNA promoter methylation of MGMT/APNG decreased in resistance cells, and increased in sensitive cells. The results agreed with the opposite trends in cellular mRNA level changes (**Supplementary Fig. 11**). For each cell line, >12 clones were bisulfite-sequenced for each condition and the data are shown as mean ± s.d. Significance was determined with an unpaired two-tailed *t*-test (** $P < 0.005$, *** $P < 0.0005$, ns $P > 0.05$).

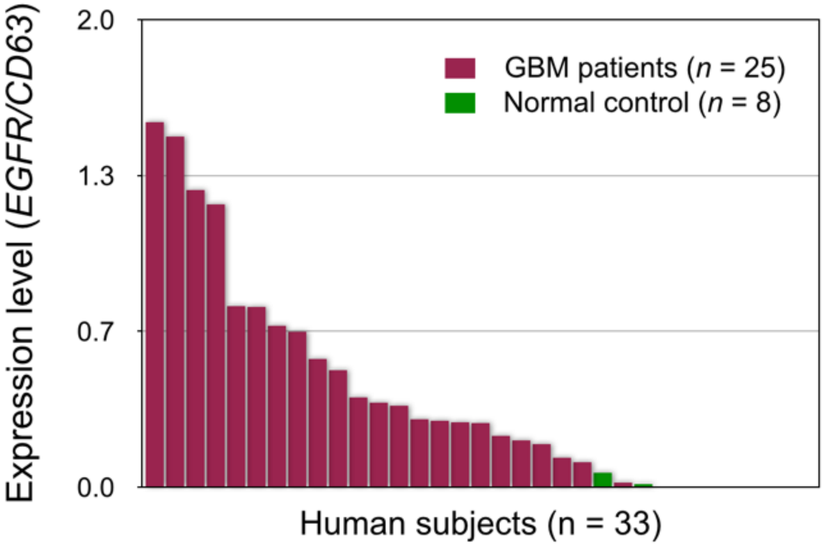

Supplementary Figure 13. Expression levels of EGFR proteins in exosomes. In a preliminary study involving clinical samples from an additional 25 GBM patients and 8 control subjects, we measured exosomal EGFR and EGFRvIII protein levels (scaled to CD63). EGFR proteins were thus used to immunomagnetically enrich GBM exosomes prior to lysis and mRNA analysis.

(a)

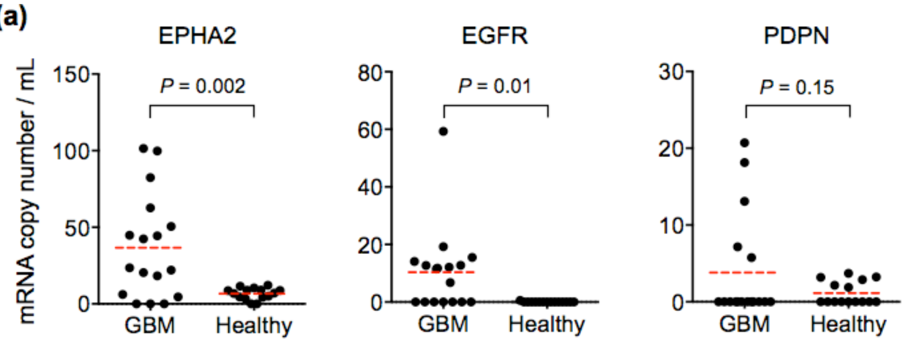

(b)

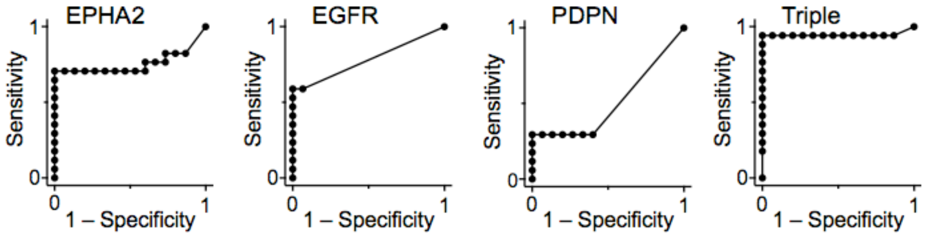

mRNA	Classification accuracy†	AUC	
		Value	Std. Err
EPHA2	84.400%	0.757	0.096
EGFR	78.100%	0.78	0.08
PDPN	43.800%	0.51	0.11
Triple markers‡	90.600%	0.945	0.054

† From the classification table of logistic regression (cutoff value, 0.5)

‡ -3.52 + 0.73 × EGFR + 0.14 × EPHA2 + 0.49 × PDPN

Supplementary Figure 14. Expression of EPHA2, EGFR, and PDPN mRNA in serum samples. (a) The exosomal mRNA levels of EPHA2 and EGFR were significantly higher in GBM patients (n = 17) than in healthy controls (n = 15), whereas the PDPN level was not statistically different among the two groups. Dotted lines indicate mean values. **(b)** The measured mRNA values were analyzed via logistic regression, using the disease state (i.e., GBM vs healthy) as a dichotomous classifier. With a single mRNA marker alone, the classification accuracy was up to 84.4%. When all three markers were used in the regression, the accuracy improved to 90.6%. The receiver-operation-characteristic curves (top) was constructed based on the predicted probability from the regression, and the area-under-curve (AUC) was calculated.

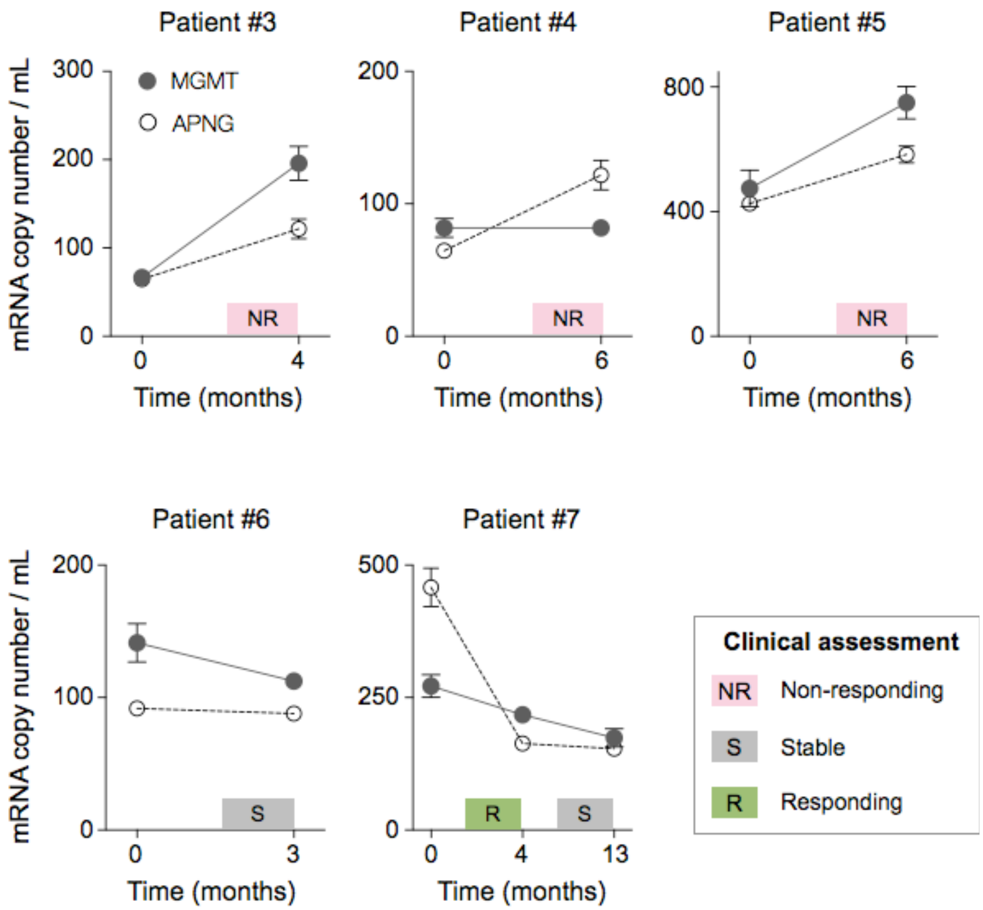

Supplementary Figure 15. Longitudinal monitoring of exosomal MGMT and APNG mRNA in GBM patients. All clinical assessments were based on radiological findings, clinical examination and lab values. While baseline levels of either MGMT and/or APNG varied across patients and were not necessarily predictive of clinical treatment response, temporal mRNA changes correlated well with treatment outcomes. All measurements were performed in triplicate and the data are shown as mean ± s.d.

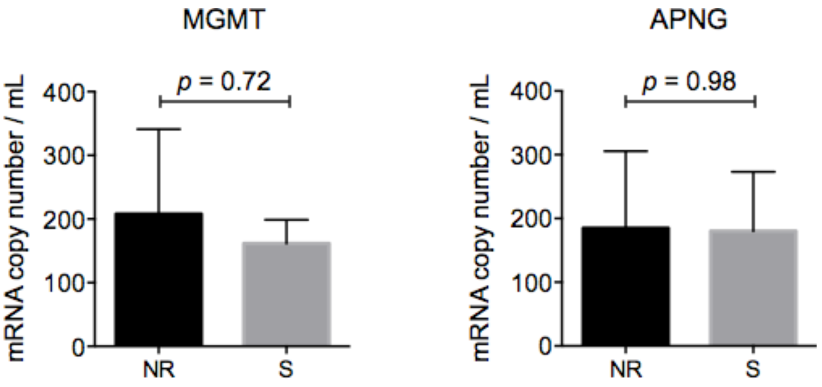

Supplementary Figure 16. Correlation between initial mRNA levels and final clinical outcomes. For both MGMT (left) and APNG (right), their levels were not significantly different (unpaired two-tailed *t*-test) between non-responding (NR, $n = 3$) and stable (S, $n = 4$) patients. The data are shown as mean ± s.d.
